# *A First-Class Degrader Candidate Targeting Both* KRAS G12D and G12V Mediated by CANDDY Technology Independent of Ubiquitination

**DOI:** 10.3390/molecules28145600

**Published:** 2023-07-24

**Authors:** Etsuko Miyamoto-Sato, Satoshi Imanishi, Lijuan Huang, Shoko Itakura, Yoichi Iwasaki, Masamichi Ishizaka

**Affiliations:** 1 R&D Department, FuturedMe Inc., 2-3-11 Honcho, Nihonbashi, Chuo-ku, Tokyo 103-0023, Tokyo, Japan; 2 Faculty of Pharmaceutical Sciences, Tokyo University of Science, 2641 Yamazaki, Noda 278-0022, Chiba, Japan; 3 Graduate School of Biological Sciences, Tokyo University of Science, 2641 Yamazaki, Noda 278-0022, Chiba, Japan

**Keywords:** target protein degradation, undruggable target, KRAS G12D, KRAS G12V, cancer, degrader, chemical knockdown, CANDDY, molecular targeted drug

## Abstract

“Undruggable” targets such as KRAS are particularly challenging in the development of drugs. We devised a novel chemical knockdown strategy, CANDDY (Chemical knockdown with Affinity aNd Degradation DYnamics) technology, which promotes protein degradation using small molecules (CANDDY molecules) that are conjugated to a degradation tag (CANDDY tag) modified from proteasome inhibitors. We demonstrated that CANDDY tags allowed for direct proteasomal target degradation independent of ubiquitination. We synthesized a KRAS-degrading CANDDY molecule, TUS-007, which induced degradation in KRAS mutants (G12D and G12V) and wild-type KRAS. We confirmed the tumor suppression effect of TUS-007 in subcutaneous xenograft models of human colon cells (KRAS G12V) with intraperitoneal administrations and in orthotopic xenograft models of human pancreatic cells (KRAS G12D) with oral administrations. Thus, CANDDY technology has the potential to therapeutically target previously undruggable proteins, providing a simpler and more practical drug targeting approach and avoiding the difficulties in matchmaking between the E3 enzyme and the target.

## 1. Introduction

The majority (75%) of disease-causing proteins are “undruggable” (i.e., difficult to inhibit with small molecules). This difficulty is reflected in the presence of smooth surfaces and a lack of deep pockets, including proteins associated with cancer drivers and many interfaces for protein–protein interactions (PPIs) [1,2,3]. RAS family members are the most challenging to inhibit with small molecules [3,4,5,6,7]. Specifically, mutations of KRAS G12C, G12D, and G12V are frequent in human cancers [7]. The RAS protein activities depend on nucleotide loading in their GTP-binding pockets. The inhibition of this pocket has been attempted for nearly 40 years. However, progress has been hindered by the exceptionally high affinity between GTP and RAS proteins [1,4,8]. While inhibitors targeting KRAS G12C have been recently clinically approved [6,9], inhibitors for KRAS G12D/V, which address a greater clinical need, remain under development. These are important targets found in 95% of pancreatic cancers and 64% of colon cancers [7,8]. Inhibitors of the PPI between RAS and Son of Sevenless 1 (SOS1) (RAS-SOS inhibitor) have been investigated. Inhibitors that directly bind to RAS are not effective due to their low affinity [5]. Thus, current inhibition technologies are not sufficient for effectively targeting undruggable proteins.

Pharmaceutical research on undruggable proteins such as RAS focuses on novel modalities instead of inhibition [10,11,12]. Proteolysis technologies using matchmakers between the target and E3 ligase or other ubiquitination-related proteins, such as molecular chaperons for inducing target degradations, are expected to be effective against undruggable proteins [13,14,15,16,17]. However, matchmaker design has been hampered by the dependency on target ubiquitination [16]. It is difficult to select a suitable E3 ligase for a target of interest because knowledge of the mechanism underlying substrate recognition by E3 ligases is limited. Even if the target has an established corresponding E3 ligase, there may be no available ligand for the E3 ligase. Moreover, target ubiquitination may not always induce proteolysis, as in the case of RAS, in which ubiquitination also regulates protein localization and activation [18]. Despite such difficulties, recently, a PROTAC of KRAS G12C [19] was developed using an effective inhibitor [9]. However, no proteolysis inducer for both KRAS G12D and G12V has been reported. The matchmaking difficulties associated with current proteolysis technologies should be eliminated [13] by various complemental and alternative tools for diverse undruggable targets (undruggable targetome).

For this study, we aimed to develop an innovative and simple set of tools (‘degraders’) mediated by CANDDY (Chemical knockdown with Affinity and Degradation DYnamics) technology. Herein, we demonstrate an approach based on multifunctional small molecules (degraders) that are constructed with target interactors and CANDDY tags. We present a proof of concept for the CANDDY strategy that not only covers druggable target degradation free from ubiquitination but also covers undruggable target degradation free from ubiquitination. We originally invented the first target degradation tool, which was mediated directly by 26S proteasome using proteasome inhibitors [20,21], before other studies reported TPD mediated by directly binding 20S proteasome [22,23]. We synthesized a CANDDY molecule, TUS-007, which induced degradation in KRAS mutants (G12D and G12V) and wild-type KRAS. We confirmed the tumor suppression effect of TUS-007 in subcutaneous xenograft models of hu-man colon cells (KRAS G12V) with intraperitoneal administrations and in orthotopic xenograft models of human pancreatic cells (KRAS G12D) with oral administrations. Notably, CANDDY technology has the potential to therapeutically target previously undruggable proteins, providing a simpler and more practical drug targeting approach and avoiding the difficulties in matchmaking between the E3 enzyme and the target.

## 2. Results

### 2.1. Proof of Concept for the CANDDY Strategy Mediated by Degradation Tags Derived from Proteasome Inhibitors

**Figure 1:**
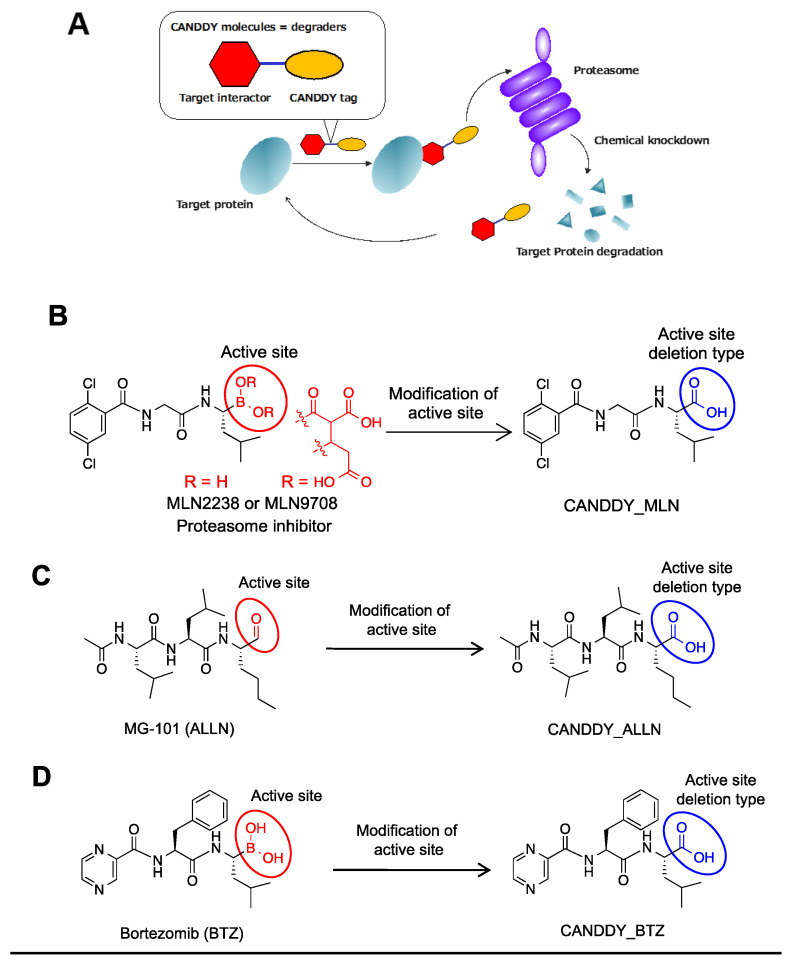
The CANDDY Strategy mediated by degradation tags derived from proteasome inhibitor. (**A**) Principle model of CANDDY technology. (**B**) CANDDY_MLN (degradation tag) chemically deletes each active site in MLN2238/9708 (MLN). (**C**) CANDDY_ALLN as a degradation tag with a chemically deleted active site from the proteasome inhibitor ALLN. (**D**) CANDDY_BTZ as a degradation tag with a chemically deleted active site from the proteasome inhibitor bortezomib (BTZ).

To address the problem of undruggable targets, we developed a simple ubiquitination-independent chemical knockdown approach named CANDDY technology (Figure 1A). CANDDY technology is based on a novel degradation tag (CANDDY tag) that has been modified from proteasome inhibitors such as MLN2238/9708 (MLN), MG-101 (ALLN), and bortezomib (BTZ), all of which have well-reported active inhibition sites [24,25] (Figure 1B–D). Our strategy is to employ proteasome inhibitors free from active inhibition sites as CANDDY tags (Figure 1B–D) for the direct proteasomal degradation of targets (Figure 1A). Even if the active inhibition site is excised, sufficient proteasome affinity could be obtained with other compound skeletons [24]. 

**Figure 2:**
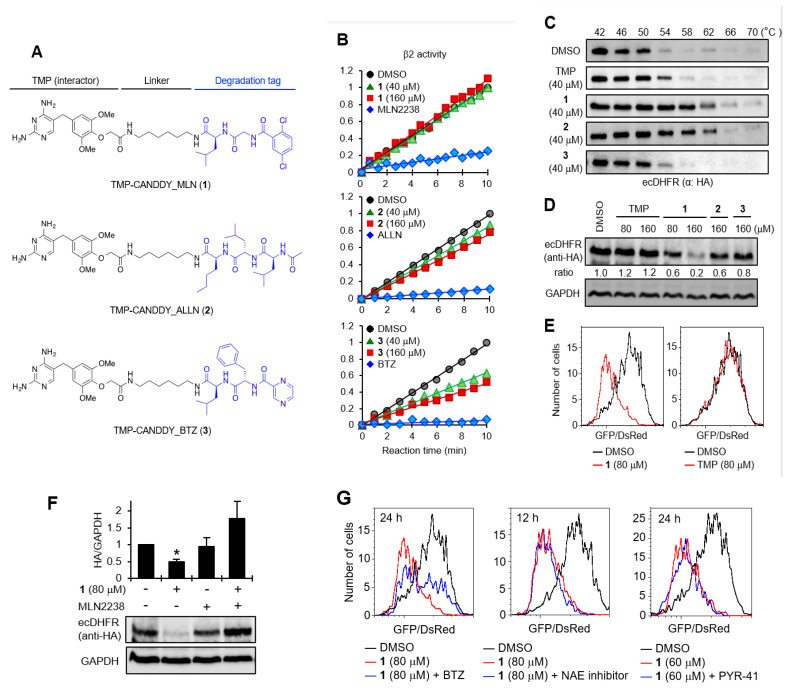
Proof of principle for the CANDDY Strategy. (**A**) Structures of degraders **1**–**3** constructed with TMP and each degradation tag (CANDDY_MLN, CANDDY_ALLN, or CANDDY_BTZ). (**B**) Comparison of proteasomal inhibitory activity of b2 by **1**–**3** and each proteasome inhibitor. (**C**) Comparison of target-binding affinity of ecDHFR and **1**–**3** by CETSA. ecDHFR-hemagglutinin (HA)-green fluorescent protein (GFP)-expressing HeLa cells were treated with **1**–**3** for 3 h. Cell extraction was treated with heat for 3 min and then analyzed via immunoblotting. (**D**) Degradation rates of ecDHFR by **1**–**3**. The same procedure as in (**E**) was performed except for 24 h. (**E**) ecDHFR degradation by **1** for 24 h. The ratio of ecDHFR-HA-GFP/DsRed was detected via flow cytometry. (**F**) Rescue of ecDHFR degradation by the proteasome inhibitor MLN2238. ecDHFR-HA-GFP-expressing HeLa cells were co-treated with **1** and MLN2238 (1.5 mM) for 24 h and then analyzed via immunoblotting (mean ± SEM; *n* = 3–4). * *p* < 0.05 (Student’s *t* test). (**G**) Ubiquitination-independent proteasomal ecDHFR degradation. ecDHFR-HA-GFP- and DsRed-expressing HeLa cells were co-treated with **1** and BTZ (proteasome inhibitor, 1.5 mM), NAE inhibitor (E3 inhibitor, 1 mM), or PYR-41 (E1 inhibitor, 10 mM), and then analyzed via flow cytometry.

**Figure 3:**
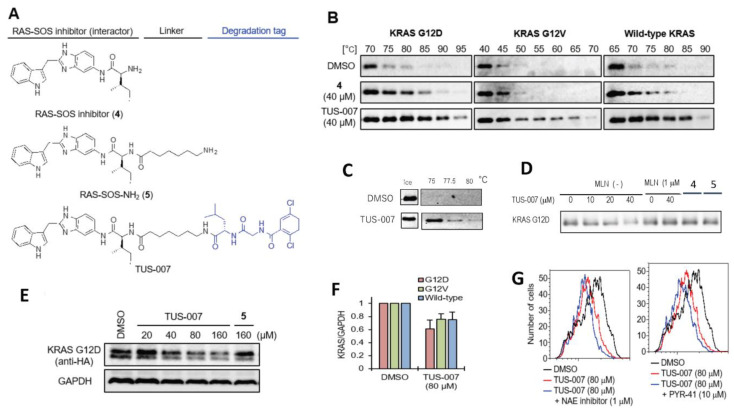
RAS Chemical Knockdown in vitro. (**A**) Structures of RAS-SOS inhibitor (**4**), RAS-SOS-NH_2_ (**5**), and TUS-007 as a RAS degrader. (**B**,**C**) Assessing the binding affinity of TUS-007 to targets KRAS (G12D, G12V, or wild-type) (**B**) and proteasome β5 (**C**) using CETSA. Recombinant KRAS or PSMB5 was treated with vehicle, **4**, or TUS-007 for 30 min. The mixture was treated with heat and then analyzed via immunoblotting. (**E**) Degradation of KRAS G12D, KRAS G12V, and wild-type KRAS proteins by TUS-007. KRAS-HA-GFP and DsRed-expressing HeLa cells were treated and analyzed via immunoblotting. (**F**) The same procedure was performed as in (**D**) (mean ± SEM; *n* = 3) except for 48 h. (**G**) Ubiquitination-independent proteasomal KRAS G12D degradation. KRAS G12D-HA-GFP and DsRed-expressing HeLa cells were co-treated with TUS-007 and the NAE inhibitor (E3 inhibitor, 1 mM) or PYR-41 (E1 inhibitor, 10 mM) and then analyzed via flow cytometry.

To demonstrate the concept of CANDDY technology, we first designed and synthesized CANDDY molecules **1**–**3** (Figure 2A), which were constructed with three different CANDDY tags: CANDDY_MLN (Figure 1B), CANDDY_ALLN (Figure 1C), and CANDDY_BTZ (Figure 1D). CANDDY molecules **1**–**3** (Figure 2A) were constructed with a target interactor, trimethoprim (TMP), which, as a druggable target, is a specific noncovalent inhibitor of *Escherichia coli* dihydrofolate reductase (ecDHFR) [26]. Proteasomes have three distinct cleavage preferences, namely chymotryptic, tryptic, and caspase-like activities, which can roughly be assigned to the subunits β5, β2, and β1, respectively [20]. Since we employed proteasome inhibitors by deleting active inhibition sites chemically, as expected, **1**–**3** had lower inhibitory activity for the subunits β5, β2, and β1 (Figure 2B and Figure S1A,B). TMP-CANDDY_MLN (**1**) showed almost no proteasomal inhibitory activity even at high concentrations. Notably, we obtained the target affinities of **1**–**3** for ecDHFR, which were not only lower (Figure 2C, **3**) but also unexpectedly more efficient (Figure 2C, **1** and **2**) compared with that of TMP (target interactor) itself in a cellular thermal shift assay (CETSA) [27]. These results suggest that the addition of a degradation-inducing tag can alter the properties of a compound. For example, we believe that it may change factors such as solubility, membrane permeability, and the overall affinity of the compound.

Next, the target-degrading efficiencies of **1**–**3** with different CANDDY tags were assessed in target (ecDHFR)-expressing HeLa cells via immunoblotting (Figure 2D) and flow cytometry (Figure 2E and Figure S2). The target degradation experiments showed that CANDDY molecules induced dose-dependent ecDHFR degradation (Figure 2D and Figure S2A), whereas TMP itself did not (Figure 2D,E). We performed additional experiments to confirm the rescue of ecDHFR degradation by CANDDY tags (Figure 2F and Figure S2B) and time-dependent ecDHFR degradation by **1** (Figure S2C). These results showed that any CANDDY tags (Figure 1B–D) induce the direct proteasomal degradation of ecDHFR and that **1** had the highest target degradation efficiency. Desirable factors to consider when selecting the best CANDDY tag include an efficient target affinity (Figure 2C) and a lower capacity for the proteasomal inhibition of CANDDY molecules (Figure 2B and Figure S1).

To confirm our ubiquitin-independent target degradation via CANDDY molecules approach, we evaluated the effect of ubiquitin-activating enzyme (E1) and E3 on-target degradation inhibitors. Co-treatment with **1** and the inhibitors of E1 or E3 did not inhibit ecDHFR degradation, in contrast to treatment with CANDDY tag (Figure 2G). These data suggest that CANDDY molecules allowed for direct ubiquitination-free proteasomal degradation of the target protein.

### 2.2. Chemical Knockdown of KRAS Mutants In Vitro by TUS-007

To further assess the general versatility of CANDDY technology, we applied it to the notoriously undruggable target RAS. We designed and synthesized a RAS degrader, TUS-007 (Figure 3A), which was constructed with RAS-SOS-NH_2_ (Figure 3A (**5**)), a derivative of a RAS-SOS inhibitor (RAS-SOS interactor) (Figure 3A (**4**)) [5], and CANDDY_MLN, which exhibited the best performance as a degradation tag (Figure 1B). Figure 3A (**4**) shows a RAS-SOS inhibitor that binds to KRAS and blocks binding to SOS, thereby causing the inhibition of SOS-mediated nucleotide exchange [5]. The binding partners of the RAS-SOS inhibitor shown in Figure 3A (**4**) include KRAS mutants KRAS G12V and KRAS G12D (*K*_D_ = 190 μM) [5]. We confirmed that TUS-007 did not exert effective inhibitory activity on the β5/β2/β1 of the proteasome (Figure S3A). Thermal shift experiments showed a higher affinity of TUS-007 for KRAS (G12D, G12V, or wild-type) compared to (**4**) (target interactor) in Figure 3B. Furthermore, TUS-007 with CANDDY tag binds to proteasome β5, unlike the interactor in Figure 3C despite not exerting efficient inhibitory activity on the proteasome (Figure S3A). Importantly, our results demonstrate that TUS-007, not Figure 3A ((**4**) and (**5**)), induces the degradation of the target protein KRAS G12D, even in a cell-free system consisting of only proteasome and the target protein (Figure 3D). These findings highlight the ability of the CANDDY tag to trigger degradation independently of ubiquitination processes.

We successfully achieved the chemical knockdown of exogenously expressed wild-type KRAS and mutant KRAS (G12V, G12D) using TUS-007, even at concentrations lower than the KD value (=190 uM) (Figure 3D–F). Previous studies have demonstrated that target degradation can occur at one-tenth of the inhibitor’s KD value [28]. Flow cytometry (Figure S3B) and immunoblotting (Figure 3E) revealed dose-dependent KRAS degradation upon TUS-007 treatment. However, Figure 3A (**5**) showed no degradation (Figure 3E), indicating that TUS-007 without the CANDDY tag failed to induce target degradation, which is consistent with the results of cell-free degradation (Figure 3D). Encouragingly, TUS-007-induced KRAS degradation could be rescued by proteasome inhibitors themselves (Figure S3C). Moreover, in contrast to treatment with proteasome inhibitors (Figure S3C), the co-treatment of TUS-007 with E1, E3, HSP90, or HSP70 inhibitors did not inhibit KRAS degradation (Figure 3G and Figure S3D). These findings suggest that TUS-007 enables the direct proteasomal degradation of undruggable target proteins without ubiquitination. Importantly, our results highlight the potential of CANDDY technology to target challenging undruggable proteins such as KRAS and demonstrate that TUS-007 can induce the targeted degradation of both KRAS mutants (G12D and G12V) independent of ubiquitination.

### 2.3. Apoptosis Induction and Tumor Suppression in Cetuximab-Resistant Human Colon Carcinoma Cells by TUS-007

**Figure 4:**
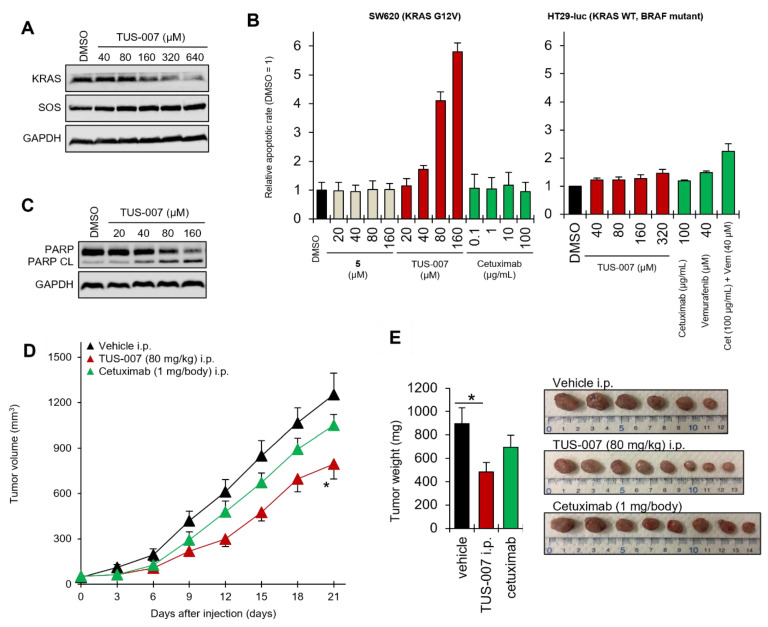
Apoptosis induction and tumor suppression in colon cancer with SW620 cells. (**A**) KRAS G12V degradation. SW620 cells were treated with TUS-007 for 48 h and then analyzed via immunoblotting. SOS was detected as a negative control. (**B**) Apoptosis-inducing activity of TUS-007. SW620 cells were treated with **5** (negative control), TUS-007, and cetuximab for 24 h and then analyzed via flow cytometry (mean ± SEM; *n* = 2). See also Figure S4A. (**C**) Immunoblotting for PARP cleavage. SW620 cells were treated with TUS-007 for 48 h. (**D**) Volume of tumors from a subcutaneous SW620 xenograft model treated with vehicle (*n* = 6), TUS-007 (*n* = 8), or cetuximab (*n* = 8) via intraperitoneal injection every three days (mean ± SEM). * *p* < 0.05 (Student’s *t* test). See also Figure S4B. (**E**) Tumor weight at day 21 in Figure 5D (mean ± SEM; *n* = 6–8). * *p* < 0.05 (Student’s *t* test).

KRAS mutations as biomarkers predict resistance to drugs such as cetuximab in colorectal cancer. Cetuximab, a monoclonal antibody that binds the extracellular domain of epidermal growth factor receptor (EGFR), is effective in KRAS wild-type metastatic colorectal cancers but not in KRAS mutations [29]. We treated SW620 human colon cancer cells (homozygous KRAS G12V) to assess the effect of TUS-007 on cetuximab-resistant cancer cells and demonstrated dose-dependent KRAS G12V degradation (Figure 4A). Here, the SOS protein was not decreased by TUS-007, which is in agreement with a previous study on the binding partners of Figure 3A (**4**) (KRAS G12V but not SOS) [5]. Therefore, TUS-007 can specifically induce RAS protein degradation in the RAS-SOS complex. Next, we found a drastic dose-dependent apoptotic response following TUS-007 administration due to KRAS G12V degradation, whereas there was no response to cetuximab, as measured by Annexin-V staining (flow cytometry) (Figure 4B). We also confirmed a dose-dependent apoptotic response to TUS-007, as measured by poly (ADP-ribose) polymerase (PARP) cleavage (immunoblotting) (Figure 4C). On the other hand, we showed that TUS-007 does not induce apoptosis against a RAS-independent BRAF mutant (human colon HT-29) cell line (Figure 4B). As expected, TUS-007 could respond selectively to cetuximab-resistant SW620 cancer cells with KRAS (G12V) as a driver.

Additionally, to evaluate the effect of tumor suppression using TUS-007 in a subcutaneous xenograft model of SW620-luc human colon cells (homozygous KRAS G12V), tumor-bearing nude mice were administered TUS-007 (80 mg/kg), cetuximab (1 mg/body), or the vehicle control via intraperitoneal injection. The administration of TUS-007 significantly attenuated tumor progression compared with the vehicle, as determined by serial volumetric measurements (Figure 4D); postmortem tumor weight was also decreased (Figure 4E). In contrast, the administration of cetuximab resulted in no significant differences in tumor volume or weight compared with the vehicle (Figure 4D,E). Additionally, we detected KRAS G12V degradation in subcutaneously transplanted tumors treated with TUS-007, as measured via immunoblotting at 21 days after injection (Figure S4A). The mice did not exhibit any significant decreases in body weight during TUS-007 treatment (Figure S4B) without significant toxicity. TUS-007 shows promise in the treatment of drug-resistant KRAS-mutant tumors through direct KRAS G12V degradation.

### 2.4. Apoptosis Induction and Tumor Suppression in Human Pancreatic Carcinoma Cells by TUS-007

**Figure 5:**
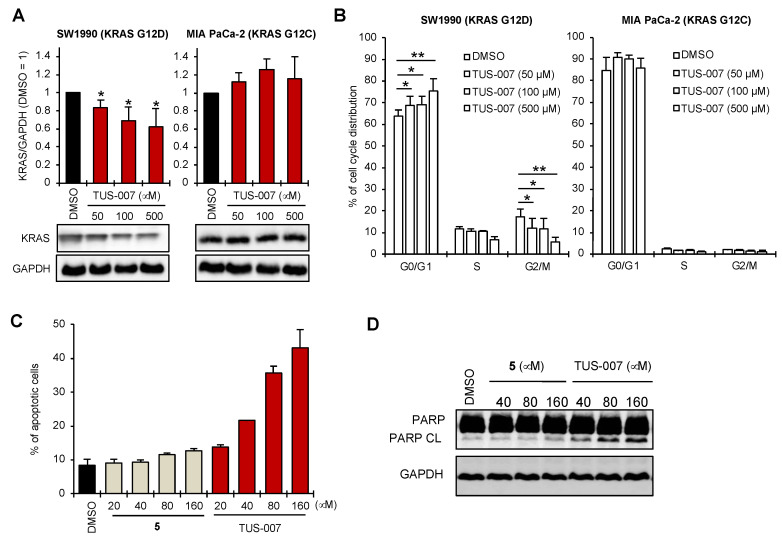
Apoptosis induction in pancreatic cancer with SW1990 cells**.** (**A**) KRAS G12D degradation by TUS-007. SW1990 and MIA PaCa-2 cells were treated with TUS-007 for 72 h and then analyzed via immunoblotting (mean ± SEM; *n* = 4). * *p* < 0.05 (Student’s *t* test). (**B**) Cell cycle analysis. SW1990 and MIA PaCa-2 cells were treated with TUS-007 for 72 h and then analyzed via flow cytometry (mean ± SEM; *n* = 5). ** *p* < 0.01, * *p* < 0.05 (Student’s *t* test). See also Figure S5. (**C**) Apoptosis-inducing activity of TUS-007. SW1990 cells were treated with **5** (negative control) and TUS-007 for 24 h and then analyzed via flow cytometry (mean ± SEM; *n* = 2). (**D**) Immunoblotting for PARP cleavage. SW1990 cells were treated with TUS-007 for 24 h.

To assess the efficacy of TUS-007 in pancreatic cancer, for which the development of an effective molecular targeted drug has thus far been unsuccessful, we assayed apoptosis and antitumor effects on SW1990 human pancreatic cancer cells (homozygous KRAS G12D). To verify RAS-driven degradation and the cause-and-effect link between RAS degradation and drug efficacy, we first observed the degradation of KRAS G12D in SW1990 cells but not KRAS G12C in MIA PaCa-2 human pancreatic cancer cells (homozygous KRAS G12C) (Figure 5A). Next, we evaluated the effect of TUS-007 on the cell cycle in SW1990 cells, and the results revealed that TUS-007 controls cell cycle suppression in a dose-dependent manner, resulting in a significant increase in G0/G1 phase and decrease in S phase and G2/M phase (Figure 5B and Figure S5). TUS-007 showed no cell cycle suppression in MIA PaCa-2 cells expressing KRAS G12C (Figure 5B and Figure S5). Accordingly, we confirmed the dose-dependent apoptotic response to TUS-007, as measured by Annexin-V staining (flow cytometry) (Figure 5D) and by PARP cleavage (immunoblotting) (Figure 5E). These observations suggest that even the moderate degradation of KRAS G12D by TUS-007 could allow for dose-dependent degradation (Figure 5A), cell cycle suppression (Figure 5B), and dose-dependent apoptotic response (Figure 5C,D) in SW1990 cancer cells. A previous study showed that a moderate decrease in the KRAS protein is enough to inhibit tumor growth via the stronger inhibition of downstream signaling [12]. In addition, a previous study using a PROTAC molecule as a degrader showed significant drug efficacy, reporting target degradation of approximately 30% [30]. 

Next, we demonstrated the antitumor efficacy of TUS-007 in a subcutaneous xenograft model of SW1990 cells (Figure S6). We confirmed tumor regression by TUS-007 administration (Figure S6A) and moderate KRAS degradation and KRAS signaling suppression in the tumor (Figure S6B). Here, we observed a decrease in both ERK (MAPK signaling) and Akt (PI3K signaling) phosphorylation upon moderate degradation of KRAS G12D by treating SW1990 cells with TUS-007. Here, this result did not show toxicity (Figure S6C) due to the suppression of dual pathways (MAPK and PI3K), as seen in many cases of preclinical and clinical trials [31]. Additionally, in normal pancreatic tissue from subcutaneously transplanted mice, TUS-007 induced the degradation of KRAS and HRAS but not NRAS, indicating it is a non-pan-RAS degrader (Figure S6D).

**Figure 6:**
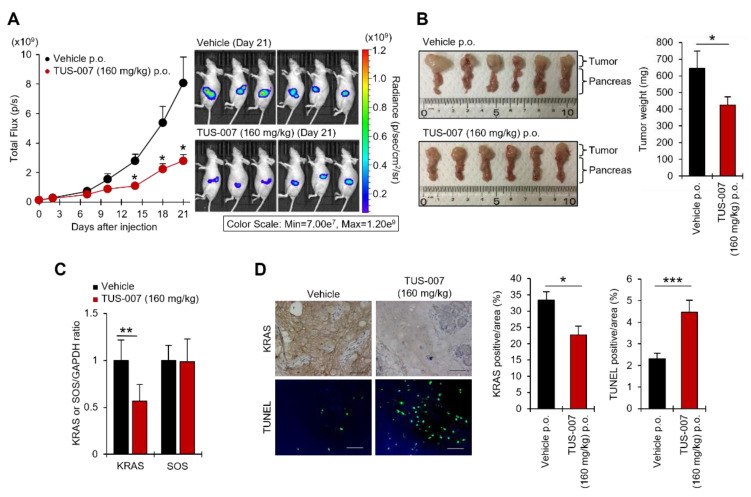
Tumor suppression in pancreatic cancer. (**A**) Tumor growth (bioluminescence) in SW1990-luc orthotopic xenograft model treated with vehicle and TUS-007 via oral administration every three days. The images of luciferase activities and total Flux were measured by in vivo imaging (mean ± SEM; *n* = 6). * *p* < 0.05 (Student’s *t* test). (**B**) Tumor weight at 21 days after injection in Figure 7A (mean ± SEM; *n* = 6). * *p* < 0.05 (Student’s *t* test). (**C**) Quantification of KRAS and SOS (negative control) proteins via immunoblotting using tumor lysates from (**B**) (mean ± SEM; *n* = 6). ** *p* < 0.01 (Student’s *t* test). See also Figure S7A. (**D**) Representative histochemical staining for KRAS and TUNEL in orthotopic tumor sections from (**D**) (left). Scale bar is 60 mm. Quantification of the histochemical staining for KRAS and TUNEL (mean ± SEM; *n* = 6) (right). *** *p* < 0.01 (Student’s *t* test).

**Figure 7:**
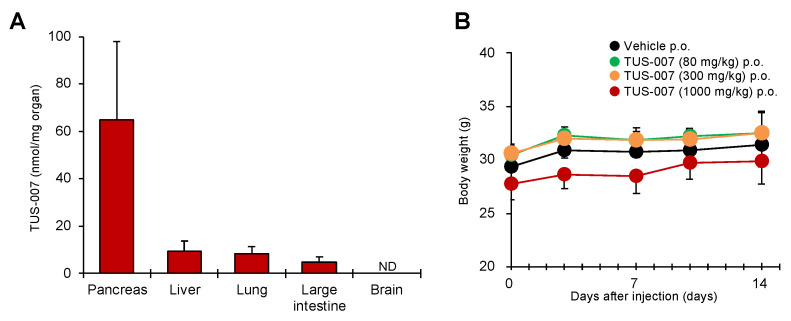
Distribution and toxicity of TUS-007. (**A**) Concentration distribution of TUS-007 in each organ of wild-type mice. Data represent mean ± SEM (*n* = 5). ND, not detected. (**B**) Toxicity test of TUS-007. Changes in body weight in mice after oral administration with the indicated concentrations (mean ± SEM; *n* = 5).

Moreover, we evaluated the viability of treating the orthotopically transplanted SW1990-luc cells of mice with TUS-007 (160 mg/kg) via oral administration (Figure 6A). Remarkably, TUS-007 also suppressed tumor volume in an orthotopically transplanted pancreatic model, as measured by an IVIS imaging system (Figure 6A). Indeed, we confirmed that the tumor weight of the TUS-007-treated group was significantly decreased (Figure 6B). Here, (**4**) or (**5**) were not used as a control for TUS-007 due of its low solubility and difficulty in administration. Additionally, we proved that the degradation of KRAS G12D can be detected in orthotopically transplanted tumors treated with TUS-007, as measured by immunoblotting (Figure 6C and Figure S7A). In our immunohistochemistry experiments, relative to the vehicle control group, statistically significant KRAS degradation and apoptosis induction (TUNEL staining) were observed in excised tumors following TUS-007 treatment (Figure 6D). Importantly, we demonstrated the suppression of a pancreatic cancer tumor that is very resistant to existing treatments through directly inducing KRAS protein degradation with a CANDDY molecule, TUS-007. Strikingly, even though drug delivery is known to be limited in the treatment of pancreatic cancer, TUS-007 functioned without exerting any toxicity and/or body weight changes (Figure S7B), even in the orthotopic-transplantation experiment with oral administration. These results are consistent with the effective delivery of TUS-007 in vivo, especially in the pancreas (Figure 7A). Moreover, we also confirmed that even a high concentration of TUS-007 (1000 mg/kg p.o.) did not affect the body weight and pathology of the mice during two weeks of observation in a single oral administration toxicity study (Figure 7C). In addition, in contrast to pan-RAS inhibition, which showed some toxicity [31], TUS-007 showed no significant toxicity because of its properties as a non-pan-RAS degrader, as demonstrated through its inability to degrade NRAS (Figure S6D).

## 3. Discussion

We provided proof of concept showing that CANDDY technology (Figure 1A) enabled the induction of proteasomal degradation through an ubiquitination-independent process in both druggable and undruggable targets (Figure 2G and Figure 3F). Considering the proteasome is capable of degrading any targets in principle, CANDDY tags modified from a proteasome inhibitor can eliminate the difficulties of matching targets and specific ubiquitination-related proteins, such as E3 ligases, in current proteins [13,14]. Accordingly, CANDDY technology could provide a simple and rational approach to induce the ubiquitination-independent chemical knockdown of even undruggable targets.

As expected, we confirmed the degradation and apoptosis of KRAS G12D and G12V in colon cancer cells (Figure 4A–C) and pancreatic cancer cells (Figure 5A,D,E), respectively. Not only in vitro but also in vivo, Even in vivo experiments, TUS-007 demonstrated anticancer effects mediated by moderate degradation (approximately 50%) of both KRAS G12V (Figure S4A) and G12D (Figure 6C). A previous study showed that a moderate decrease in the KRAS protein is enough to inhibit tumor growth via the stronger inhibition of downstream signaling [12], as we have also demonstrated (Figure S6B). Thus, our in vitro and in vivo results could be considered reasonable. Interestingly, we observed a decrease in both ERK (MAPK signaling) and Akt (PI3K signaling) phosphorylation upon the degradation of KRAS G12D via TUS-007 administration. However, this result did not show toxicity in all of the in vivo experiments performed, including a high-concentration single dose of TUS-007 (1000 mg/kg p.o.), despite the suppression of two pathways (MAPK and PI3K), as seen in many preclinical and clinical trials [32]. These results suggest that no significant toxicity may result from moderate target degradation even in the case of high concentrations. Thus, controlling the maximum degradation concentration (DCmax) is likely important for assessing toxicity and drug efficacy.

## 4. Materials and Methods

### 4.1. Synthesis of CANDDY Molecules

CANDDY molecules were synthesized independently. NMR spectra were recorded using a Bruker BioSpin AVANCE600 spectrometer, and mass spectrometry was performed on a JEOL JMS-700. Chromatography utilized silica gel and PLC silica gel 60. Reversed-phase liquid chromatography was conducted with HPLC equipment under specific conditions. Some compounds were purchased from suppliers, while others were synthesized based on previous reports. See the Supplementary Materials for details regarding the synthesis of the CANDDY molecules.

### 4.2. Evaluation of Affinity of TUS-007 for KRAS and Proteasome in a Thermal Shift Assay

To evaluate the affinity of TUS-007, a RAS-SOS inhibitor, for KRAS and PSMB5 (proteasome subunit), KRAS (100 nM) or PSMB5 (20 nM) was preincubated with TUS-007 or 10% DMSO in 75 mM phosphate buffer (pH 7.5). The preincubation was carried out for 30 min at 37 °C (wild-type and G12D KRAS, or PSMB5) or 20 min at 25 °C (G12V KRAS). Aliquots of the reaction solution were then sampled into separate microtubes and subjected to heat treatment at different temperatures: 65, 70, 80, 85, or 90 °C for wild-type KRAS; 70, 75, 80, 85, or 90 °C for G12D KRAS; 40, 45, 50, 55, or 60 °C for G12V KRAS; and 75, 77.5, or 80 °C for PSMB5. After centrifugation at 18,000× *g* for 20 min, the supernatants were analyzed via SDS-PAGE, followed by immunoblotting using an anti-KRAS antibody (1:1000, WH0003845MI, Sigma-Aldrich, St. Louis, MO, USA). Immunoreactive bands were detected using an iBright CL1000 chemiluminator (Thermo Fisher Scientific, Waltham, MA, USA) with an enhanced chemiluminescent substrate for horseradish peroxidase (HRP) detection (Merck, Darmstadt, Germany).

### 4.3. Evaluation of KRAS Degradation Induced by TUS-007 in a Cell-Free System

KRAS G12D (final concentration: 5 ng/µL) and 26S proteasome (final concentration: 8 nM) were incubated with TUS-007 (10, 20, 40 µM), RAS-SOS inhibitor (40 µM), RAS-SOS-NH2 (40 µM), or DMSO in 20 mM Tris-HCl buffer (pH 7.5) containing 20% glycerol for 3 h at 37 °C. To assess the effect of MLN on chemical knockdown, KRAS G12D and 26S proteasome were incubated with DMSO or TUS-007 40 µM in the presence or absence of MLN (1 µM) in the same condition. After centrifugation at 14,000× *g* for 10 min, the supernatants were analyzed via SDS-PAGE, followed by immunoblotting using an anti-KRAS antibody (1:1000, WH0003845MI, Sigma-Aldrich).

### 4.4. Cell Proliferation Assay by WST-8

To examine the effect of TUS-007 on cell proliferation, WST-8 assay (Cell Count Reagent SF, Nacalai Tesque) was performed. Cells, plated in 96-well plates at a density of 2 × 10^3^ cells per well, were cultured overnight and treated with the indicated concentrations of TUS-007 in serum-free medium. After 2 days of incubation at 37 °C, 100 μL of serum-containing medium was added to each well, and the plates were incubated for a further 3 or 4 days. After incubation, cell viability was quantified with the WST-8 reagent. Briefly, the culture medium was removed, and 110 μL of growth medium and WST-8 was mixed at a ratio of 1:10 and added to each well. After the cells had been incubated in 5% CO_2_ at 37 °C for 1–2 h, absorbance at 450 nm was measured.

### 4.5. KRAS Degradation Assays of TUS-007 by Western Blotting

SW620 (purchased from JCRB) and SW1990 (purchased from ATCC) cells were treated with 1% DMSO in serum-free medium or TUS-007 (100% DMSO stock solution) for 48 h (SW620 cells) or 72 h (SW1990 and MIA PaCa-2 cells). The cells were lysed using RIPA buffer (Sigma Aldrich). The precipitates were separated from the soluble fraction by centrifugation at 18,000× *g* for 20 min, and the supernatants were analyzed via Western blotting. Mouse monoclonal antibodies against KRAS (1:1000; WH0003845MI, Sigma), SOS (1:500; sc-17793, Santa Cruz Biotechnology, Inc., Dallas, TX, USA) and GAPDH (1:20,000; sc-32233, Santa Cruz Biotechnology, Inc.) were used for reactions.

### 4.6. Analysis of Cell Cycle Distribution

SW1990 and MIA PaCa-2 cells were plated onto 6-well plates at a density of 3 × 10^5^ cells per well and cultured overnight, then treated with indicated concentrations of TUS-007 in serum-free medium for 72 h. Following incubation, the cells were collected, and 1 μL of Vybrant DyeCycle™ Orange (Invitrogen, Waltham, MA, USA) was added to 1 mL of cell suspension in growth medium and incubated for 30 min at 37 °C. Cell cycle analysis was performed by FACS Calibur (Becton-Dickinson, Heidelberg, Germany) with FL2. Data were analyzed with FlowJo software v10 (Celeza, Switzerland).

### 4.7. KRAS Degradation Assays by Enzyme Linked Immunosorbent Assay (ELISA)

The expression levels of KRAS in SW1990 were also determined via ELISA (Human GTPase kRas ELISA kit; CUSABIO TECHNOLOGY LLC). Cells were treated with the indicated concentrations of TUS-007 for 24 h, then lysed with Cell Extraction buffer (Invitrogen). The assay was performed according to the manufacturer’s instructions.

### 4.8. Analysis of TUS-007-Induced Apoptosis by Western Blotting

The SW1990 cells were treated with 1% DMSO in serum-free medium or TUS-007 for 3 h. The cells were lysed using RIPA buffer (Sigma Aldrich) in the absence of protease and phosphatase inhibitors (Nacalai Tesque, Inc., Kyoto, Japan). The precipitates were separated from the soluble fraction by centrifugation at 18,000× *g* for 20 min. The supernatants were analyzed via Western blotting. Rabbit monoclonal antibodies against ERK, p-ERK, Akt, and p-Akt (1:1000; Cell Signaling Technology, Inc., Danvers, MA, USA) were used.

### 4.9. Analysis of TUS-007-Induced Apoptotic Cells by Flow Cytometry

To assess the effect of CANDDY molecules on apoptosis, SW620, HT29-Luc, and SW1990 cells were seeded in 24-well plates followed by the addition of compounds at the indicated concentrations. After 24 or 48 h, the cells were harvested using trypsin, washed with PBS, and pelleted at 110× *g* for 5 min. The cells were re-suspended in 85 mL binding buffer (Annexin V-FITC Kit, Medical & Biological Laboratories Co., Ltd., Tokyo, Japan). To each sample, 10 mL Annexin V-FITC and 5 mL propidium iodide were added for staining at room temperature for 15 min in the dark. After incubation, 400 mL binding buffer was added to the samples on ice. The cells were then analyzed by the BD FACSCanto II. Data were analyzed using FlowJo software (Way Ashland, OR, USA).

### 4.10. Antitumor Evaluation of TUS-007 in a Subcutaneous Xenograft Model

SW620 (3 × 10^7^ cells/mL) and SW1990 cells (5 × 10^7^ cells/mL) were suspended in PBS, and then 100 μL of suspension was transplanted subcutaneously into the right flank of BALB/cA-nu/nu mice using a 26 g syringe. The tumor volumes were calculated according to the formula of length × width^2^ × 0.5. When the tumor volumes reached around 100 mm^3^, the mice were randomly sorted into each group. TUS-007 was dissolved in DMSO and subsequently dilution with 20% PEG400/Tween80 (ratio of 1/1) in saline to the final concentration of 10% DMSO. The TUS-007 solution was injected intraperitoneally into the tumor-bearing mice at 80 mg/kg every three days. Cetuximab was intraperitoneally injected at 1 mg/body every three days.

### 4.11. Antitumor Evaluation of TUS-007 in an Orthotopic Xenograft Model

pGL4.51 (Luc2/CMV/Neo) vector (Promega, Madison, WI, USA) was transfected into the SW1990 cells. The SW1990 cells stably expressing luciferase (SW1990-luc) were established via selection with G418. SW1990-luc cells were mixed with growth factor-reduced Matrigel (BD Biosciences, Franklin Lakes, NJ, USA) at a ratio of 1:1 (*v*/*v*) to a final density of 1.5 × 10^4^ cells/μL on ice. Then, 10 μL of cell suspension was directly injected into the pancreas of BALB/cA-nu/nu mice using a 27 g syringe. Three days after transplantation, treatment with TUS-007 commenced; TUS-007 was suspended in 0.5% (*w*/*v*) carboxymethyl cellulose (CMC) and administered orally at 160 mg/kg every three days. Tumor luciferase activity was monitored by the corresponding bioluminescent intensity. The mice were injected subcutaneously with 50 μL D-Luciferin (30 mg/mL in saline) (Promega, Madison, WI, USA). Under anesthesia with isoflurane (Wako, Tokyo, Japan), bioluminescent images were obtained 20 min after substance injection using the IVIS Lumina LT (Perkin Elmer, Waltham, MA, USA) with an exposure time of 15 s.

### 4.12. KRAS Degradation Analysis of TUS-007 in Xenograft Tumors

The expression of KRAS in xenograft tumors was evaluated via immunohistochemistry and Western blot analysis. The tumor tissue was harvested from subcutaneous xenograft mice on the last day of antitumor evaluation. In the case of orthotopic xenograft mice, the tumor tissue was harvested at the next day of the administration for three consecutive days from Day 21. One part of the tumor was fixed in 4% paraformaldehyde and embedded in paraffin and then cut into 5 mm-thick sections. The deparaffinized sections were incubated in 0.3% hydrogen peroxidase (H_2_O_2_) in methanol for 30 min, and this was followed by antigen retrieval using 10 mM citrate buffer (pH 6.0) at 121 °C for 20 min. The tumor tissues were blocked with 1% bovine serum albumin (BSA) in PBS for 30 min and stained with primary antibodies against human KRAS (1:200; 12063-1-AP, proteintech, Rosemont, IL, USA) diluted in 1% BSA. Next, the tissues were incubated with the HRP-labeled secondary antibodies (1:600; ab6721, Abcam, Cambridge, UK) in 1% BSA for 30 min at room temperature. The sections were counterstained with hematoxylin and then visualized by DAB (1.02924.0001, Merck, Darmstadt, Germany). The digital images were obtained by microscopy (BZ-9000, KIYENC, Osaka, Japan). The individual area of each KRAS stain was identified and measured using image J software v1.53, and the perimeter to area ratio (KRAS positive area/section) was calculated. Altogether, 30 images were analyzed in six tumor sections from each group. The remaining part of each tumor sample was homogenized in TKM buffer for Western blot analysis as described above. Mouse monoclonal antibodies against KRAS (1:1000; WH0003845MI, Sigma), SOS (1:500; sc-17793, Santa Cruz Biotechnology, Inc.), and GAPDH (1:20,000; sc-32233, Santa Cruz Biotechnology, Inc.) and Rabbit antibodies against p-Erk1/2 (phospho-p44/42 MAPK) (1:1000; #4370, Cell Signaling, Danvers, MA, USA), Erk1/2 (p44/42 MAPK) (1:1000; #4695, Cell Signaling), p-Akt (1:2000; #4060, Cell Signaling), and Akt (1:2000; #4691, Cell Signaling) were used for reactions.

### 4.13. RAS Family Degradation Assay of TUS-007 in Normal Tissue of Mice

Normal pancreatic tissues from subcutaneously transplanted mice were homogenized in T-PER^TM^ tissue protein extraction reagent (Thermo Scientific, Waltham, MA, USA) containing protease inhibitor cocktail (EDTA-free, Roche, Basel, Switzerland). Lysates were centrifuged at 18,000× *g* for 30 min, and then the supernatants were analyzed by Western blotting. Mouse monoclonal antibodies against KRAS (1:1000; WH0003845MI, Sigma) and GAPDH (1:10,000; sc-32233, Santa Cruz Biotechnology, Inc.) and rabbit polyclonal antibodies against HRAS (1:1000; 18295-1-AP, Proteintech) and NRAS (1:1000; 10724-1-AP, Proteintech) were used for the reactions. 

### 4.14. TUNEL Assay of Xenograft Tumors Treated with TUS-007

DNA fragmentation in tumor tissue was evaluated via TdT-mediated dUTP Nick-End Labeling (TUNEL) assay. The paraffin-embedded tissue sections (5 mm) were deparaffinized. The TUNEL reaction was performed using the DeadEnd Fluorometric TUNEL System (Promega, Madison, WI, USA) according to the manufacturer’s instructions. The fluorescent images were acquired using a fluorescence microscope (BZ-9000, KIYENC). TUNEL-positive cells were identified and calculated by using image J software. In total, 30 images were analyzed in six tumor sections from each group.

### 4.15. Pharmacokinetic Analysis

TUS-007 was administrated to ICR mice by oral gavage (80 mg/kg), intraperitoneal injection (80 mg/kg), and intravenous injection (20 mg/kg). The blood was collected by cardiocentesis under isoflurane anesthesia at the indicated time. The blood sample was added with 10 volumes of acetonitrile and centrifuged at 2700× *g* for 20 min. The supernatant was evaporated and subsequently subjected to reconstitution in mobile phase. Then, TUS-007 was analyzed using high-performance liquid chromatography (HPLC) (EXTREMA, JASCO Corp., Tokyo, Japan). The HPLC runs were performed using a column of Inertsil ODS-4 (250 × 4.6 mm, GL Science Inc., Tokyo, Japan) with the isocratic elution mode with 0.2% trifluoroacetic acid (TFA) in 550:450 (*v*/*v*) acetonitrile/water mobile phase at a flow rate of 1.0 mL/min. The peak blood concentrations (C_max_) and the time of maximum concentration (T_max_) were directly obtained from the blood concentration–time profiles. The area under the blood concentration–time curve (AUC) and mean retention time (MRT) were calculated using a moment analysis method. These parameters were calculated by integration to infinite time.

### 4.16. Tissue Distribution of TUS-007 

To evaluate the tissue distribution of TUS-007 in vivo, the organs of ICR mice in the PK analysis were used. A total of 40–80 mg of each organ was homogenized in 0.6 mL of tissue lysis buffer (50 mM Tris-HCl pH 8.0, 20 mM EDTA, 10 mM NaCl, 1% SDS) by Micro Smash MS-100 using stainless (5.5φ) and zirconia (1.0φ) beads (TOMY SEIKO CO., LTD., Tokyo, Japan). Lysates were centrifuged at 18,000× *g* for 10 min, and 2.25 mL of methanol/chloroform (ratio of 2/1) was added to the supernatants, which were then left to stand for 30 min at room temperature. Then, 0.75 mL of chloroform and 0.75 mL of distilled water were added and then centrifuged at 1500× *g* for 15 min. The layer of chloroform was dried under reduced pressure and redissolved with the mobile phase. TUS-007 was analyzed by HPLC (Alliance e2695, Waters Corporation., MA, USA) using a column of COSMOSIL^®^ C18-AR-II (150 × 4.6 mm, Nacalai Tesque., Kyoto, Japan) with the isocratic elution mode with 0.1% trifluoroacetic acid (TFA) in 50:50 (*v*/*v*) acetonitrile/water mobile phase at a flow rate of 1.0 mL/min.

### 4.17. In Vivo Toxicity Test of TUS-007

TUS-007 was reconstituted in 10% DMSO diluted in 0.5% CMC solution, and a vehicle solution containing 10% DMSO in 0.5% CMC was used as a control. ICR mice were treated by oral gavage using a feeding needle with TUS-007 at 80, 300, and 1000 mg/kg (*n* = 5/group), and their body weights were measured twice-weekly. Two weeks later, the mice were euthanized, and the organs in the abdominal cavity were observed and evaluated. The spleens revealed no decrease in size with this treatment compared with the controls.

## 5. Conclusions

As of yet, no drugs have been reported to effectively inhibit or degrade both KRAS G12D and G12V. Additionally, the field of proteolysis lacks substantial evidence to support its ability to target and degrade tough and valuable undruggable proteins [14].Notably, despite the use of a poor inhibitor as an interactor, TUS-007 induced the chemical knockdown of both KRAS G12D and G12V in vivo, resulting in the suppression of a pancreatic tumor even in the orthotopic xenograft model involving oral administration (Figure 6). This result is particularly significant considering the lack of effective drugs for pancreatic cancer due to difficulties in compound delivery. These findings align with the successful delivery of TUS-007 to the pancreas compared to other organs (Figure 7A). Moreover, in the case of undruggable targets, it is often common to only have such weak inhibitors (5). Therefore, this time, we believe that, even with such a limited inhibitor option, obtaining a candidate to progress to animal experiments demonstrates a significant step. CANDDY technology could be applied to tough and valuable undruggable targets with no remaining effective inhibitors. A direct degradation method using proteasomes can cover the shortcomings of E3 and attract attention among researchers [33]. In the future, CANDDY has the potential to overcome the current limitations of proteolysis and expand the scope of application for degraders used in molecular targeting therapies for aggressive and life-threatening diseases associated with undruggable targets.

## Data Availability

Not applicable.

## Supplementary Materials

The following supporting information can be downloaded at: https://www.mdpi.com/article/10.3390/molecules28145600/s1. Supplemental Information includes the synthesis of CANDDY molecules and figures.
