# Supplementary material for: A First-Class Degrader Candidate Targeting Both KRAS G12D and G12V Mediated by CANDDY Technology Independent of Ubiquitination
Source: Molecules. 2023 Jul 24;28(14):5600. doi: 10.3390/molecules28145600 (PMC10386196; doi:10.3390/molecules28145600)
Supplement: Supplementary file 1 [file molecules-28-05600-s001.zip › Molecules_Supplementary Information.pdf]

Supplementary Information for "A first-in-class degrader candidate targeting both KRAS G12D and G12V mediated by CANDDY technology independent of ubiquichination"

Etsuko Miyamoto-Sato[1, 2, 3 *], Satoshi Imanishi[3], Lijuan Huang[3], Shoko Itakura[3], Yoichi Iwasaki[1], and Masamichi Ishizaka[1]

[1]R & D Department, FuturedMe Inc., 2-3-11 Honcho, Nihonbashi, Chuo-ku, Tokyo 103-0023, Japan

[2]Faculty of Pharmaceutical Sciences, Tokyo University of Science, 2641 Yamazaki, Noda, Chiba 278-0022, Japan

[3]Graduate School of Biological Sciences, Tokyo University of Science, 2641 Yamazaki, Noda, Chiba 278-0022, Japan

[*] Correspondence: etsuko.miyamoto-sato@futuredme.com and nekoneko@rs.tus.ac.jp

Synthesis of CANDDY molecules

Nuclear magnetic resonance (NMR) spectra were recorded on a Bruker BioSpin AVANCE600 spectrometer operating at 600 MHz for ^{1}H-NMR. Chemical shifts were reported in the scale relative to CD_3OD as an internal reference. Fast-atom bombardment mass spectrometry was performed on a JEOL JMS-700. Column chromatography was performed with silica gel 60 (75 mm) purchased from Nacalai Tesque, Inc. PLC silica gel 60 was purchased from Merck KGaA. Reversed phase liquid chromatography was performed with high performance liquid chromatography (HPLC) (Shimadzu Co., Ltd), with a YMC-Pack ODS-A or Inertsil ODS-4 (GL Science) (250 × 4.6 mm), an LC-6AD pump, and a RID-10A detector (Shimadzu Co., Ltd). HPLC experiment was performed with isocratic condition A ($MeCN/H_2O/TFA$, 70:30:0.1; flow rate, 1 mL/min) for compounds **1**–**3**, and condition B ($MeCN/H_2O/TFA$, 55:45:0.1; flow rate, 1 mL/min) for TUS-007. H-Gly-OtBu · HCl and 2,5-dichlorobenzoic acid were purchased from Ark Pharm. H-Leu-OtBu · HCl and H-Phe-OtBu · HCl were purchased from Watanabe Chemical Industry. Synthesis of $TMP-NH_2$ was based on a previous report (Long et al., 2011; Long et al., 2012). The synthesis of RAS-SOS inhibitor (**4**) and RAS-SOS-NH_2 (**5**) was based on a previous report (Sun et al., 2012). A part of the compound was synthesized by NARD Institute. Ltd. (Kobe, Japan).

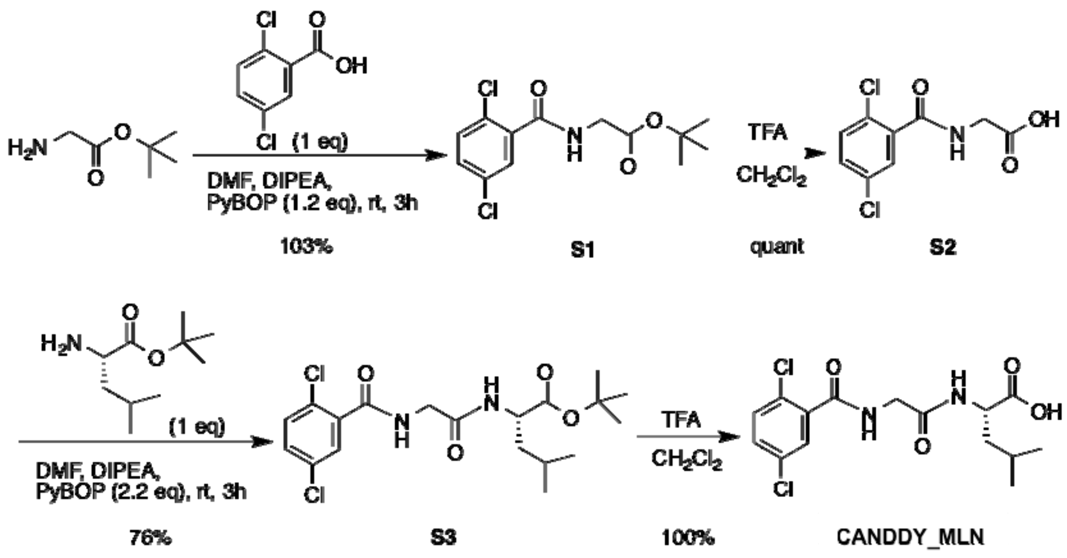

Synthesis of CANDDY_MLN

To a solution of H-Gly-OtBu · HCl (286.8 mg, 1.69 mmol) and 2,5-dichlorobenzoic acid (309.3 mg, 1.62 mmol) in dehydrated *N*,*N*-dimethylformamide (10 mL) and *N*,*N*-diisopropylethylamine (5 mL), PyBOP (1.02 g, 1.92 mmol) was added at room temperature. After stirring for 3 h at the

same temperature, a saturated aqueous solution of sodium bicarbonate was added at 0°C. The organic layer was separated and the aqueous layer was extracted with ethyl acetate/hexane (4/1). The combined organic layers were dried over anhydrous sodium sulfate and concentrated *in vacuo*. Further purification was carried out by silica gel column chromatography (eluent; hexane/chloroform = 1/1–0/1, gradient) to produce **S1** (531.0 mg, 100%). To a solution of **S1** (212.4 mg, 0.70 mmol) in dichloromethane (5 mL), trifluoroacetic acid (5 mL) was added at room temperature. After stirring for 1 h at the same temperature, the reaction mixture was concentrated *in vacuo* to produce **S2** (190.7 mg, 110%). To a solution of **S2** (190.7 mg, 0.77 mmol) and H-Leu-OtBu · HCl (175.8 mg, 0.79 mmol) in dehydrated *N*,*N*-dimethylformamide (5 mL) and *N*,*N*-diisopropylethylamine (5 mL), PyBOP (886.7 mg, 1.70 mmol) was added at room temperature. After stirring for 3 h at the same temperature, a saturated aqueous solution of sodium bicarbonate was added at 0°C. The organic layer was separated and the aqueous layer was extracted with ethyl acetate/hexane (4/1). The combined organic layers were dried over anhydrous sodium sulfate and concentrated *in vacuo*. Further purification was carried out by silica gel column chromatography (eluent: hexane/chloroform; 1/1–0/1, gradient) to produce **S3** (244.2 mg, 76%). To a solution of **S3** (240.8 mg, 0.58 mmol) in dichloromethane (5 mL), trifluoroacetic acid (5 mL) was added at room temperature. After stirring for 1 h at the same temperature, the reaction mixture was concentrated *in vacuo* to produce **CANDDY_MLN** (214.7 mg, 100%).

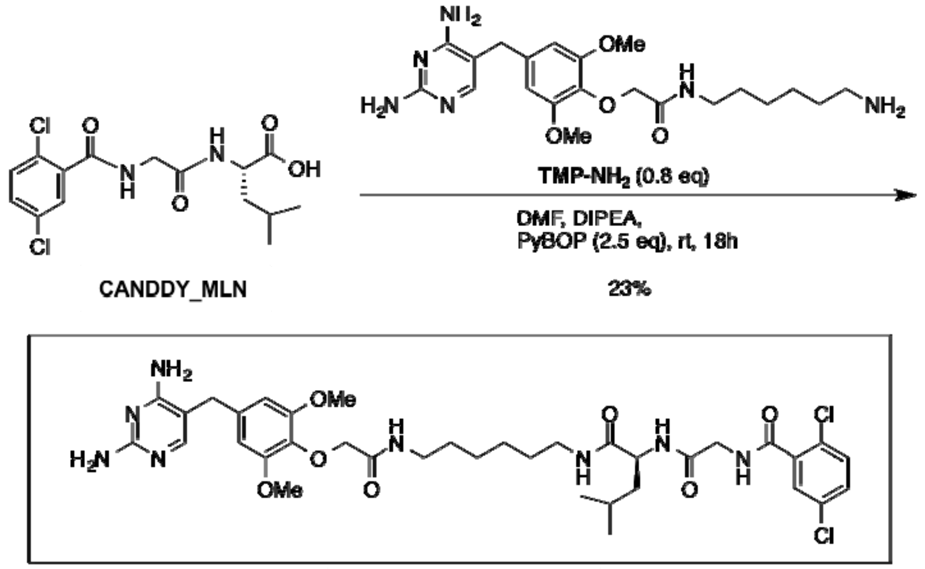

TMP-CANDDY_MLN (1)

Synthesis of TMP-CANDDY_MLN (1)

To a solution of **CANDDY_MLN** (210.3 mg, 0.58 mmol) and TMP-NH_2 (207.6 mg, 0.48 mmol) in dehydrated *N*,*N*-dimethylformamide (5 mL) and *N*,*N*-diisopropylethylamine (5 mL), PyBOP (765.2 mg, 1.47 mmol) was added at room temperature. After stirring for 18 h at the same temperature, a saturated aqueous solution of sodium bicarbonate was added at 0°C. The organic layer was separated and the aqueous layer was extracted with ethyl acetate. The combined organic layers were dried over anhydrous sodium sulfate and concentrated *in vacuo*. Further purification was carried out by silica gel column chromatography (eluent: chloroform/methanol; 20/1–4/1, gradient) to produce **TMP-CANDDY_MLN** (**1**) (102.3 mg, 23%, isolated yield). A compound for biological assays was purified by PLC (eluent: chloroform/methanol; 10/1). ^{1}H NMR (600 MHz, CD_3OD) δ 0.94 (dd, *J* = 6.6 Hz, 18.0 Hz, 6H), 1.33 (m), 1.47–1.72 (m), 3.17 (dd, *J* = 6.6 Hz, 6.6 Hz), 3.27 (dd, *J* = 6.6 Hz, 6.6 Hz), 3.65 (s, 2H), 3.82 (s, 6H), 4.38 (s, 2H), 4.39 (dd, *J* = 5.4 Hz, 9.6 Hz), 6.56 (s, 2H), 7.41–7.44 (m, 2H), 7.46 (s), 7.59 (dd, *J* = 0.6 Hz, 2.4 Hz); HRMS-FAB (m/z): $[M+H]^+$ calcd for $C_{36}H_{49}Cl_2N_8O_7$, 775.3101; found, 775.3104. HPLC: *t*R = 7.69 min, purity; 98% (λ = 254 nm).

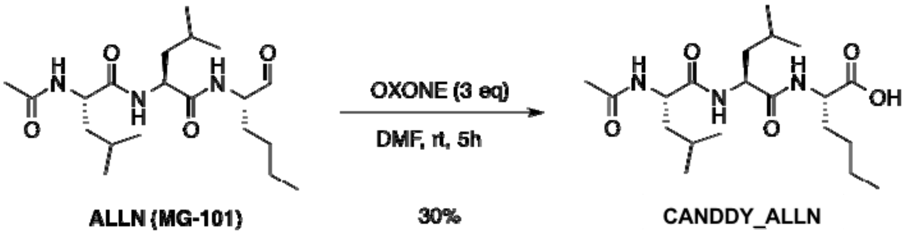

Synthesis of CANDDY_ALLN

To a solution of ALLN (87.2 mg, 0.23 mmol) in dehydrated *N*,*N*-dimethylformamide (2 mL), OXONE® (212.1 mg, 0.69 mmol) was added at room temperature. After stirring for 5 h at the same temperature, water was added at 0°C. The organic layer was separated and the aqueous layer was extracted with chloroform. The combined organic layers were dried over anhydrous sodium sulfate and concentrated *in vacuo*. Further purification was carried out by silica gel column chromatography (eluent: chloroform/methanol; 20/1–10/1, gradient) to produce **CANDDY_ALLN** (27.0 mg, 30%).

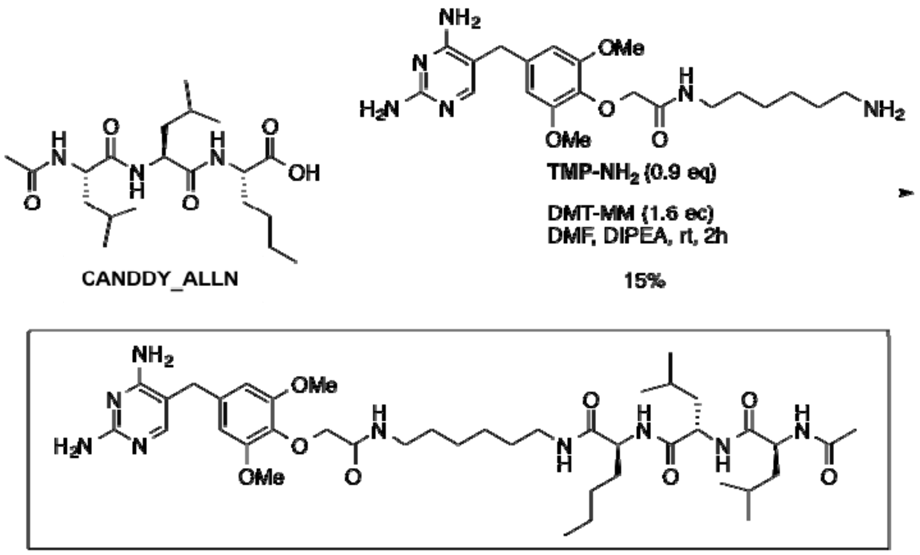

TMP-CANDDY_ALLN (2)

Synthesis of TMP-CANDDY_ALLN (2)

To a mixture of a solution of **CANDDY_ALLN** (26.8 mg, 0.067 mmol) and TMP-NH_2 (26.0 mg, 0.060 mmol) in dehydrated *N*,*N*-dimethylformamide (2 mL) and *N*,*N*-diisopropylethylamine (0.1 mL), DMT-MM (30 mg, 0.11 mmol) was added at room temperature. After stirring for 2 h at the same temperature, brine and 0.1 N hydrochloric acid were added at 0°C. The organic layer was separated and the aqueous layer was extracted with ethyl acetate. The combined organic layers were dried over anhydrous sodium sulfate and concentrated *in vacuo*. Further purification was carried out by silica gel column chromatography (eluent: chloroform/methanol; 10/1) to produce **TMP-CANDDY_ALLN** (**2**) (8.2 mg, 15%, isolated yield). A compound for biological assays was purified by PLC (eluent: chloroform/methanol; 10/1). ^{1}H NMR (600 MHz, CD_3OD) δ 0.88–0.95 (m, 15H), 1.25–1.40 (m), 1.46–1.79 (m), 3.16 (m, 2H), 3.66 (s, 2H), 3.82 (s, 6H), 4.22 (dd, *J* = 6.0 Hz, 9.0 Hz), 4.33 (dd, *J* = 7.8 Hz, 7.8 Hz), 4.37 (dd, *J* = 6.0 Hz, 9.6 Hz), 4.39 (s, 2H), 6.57 (s), 7.49 (s); HRMS-FAB (m/z): $[M+H]^+$ calcd for $C_{41}H_{68}N_9O_8$, 814.5191; found, 814.5186. HRMS-FAB (m/z): $[M+H]^+$ calcd for $C_{36}H_{49}Cl_2N_8O_7$, 775.3101; found, 775.3104. HPLC: *t*R = 7.66 min, purity; 100% (λ = 254 nm).

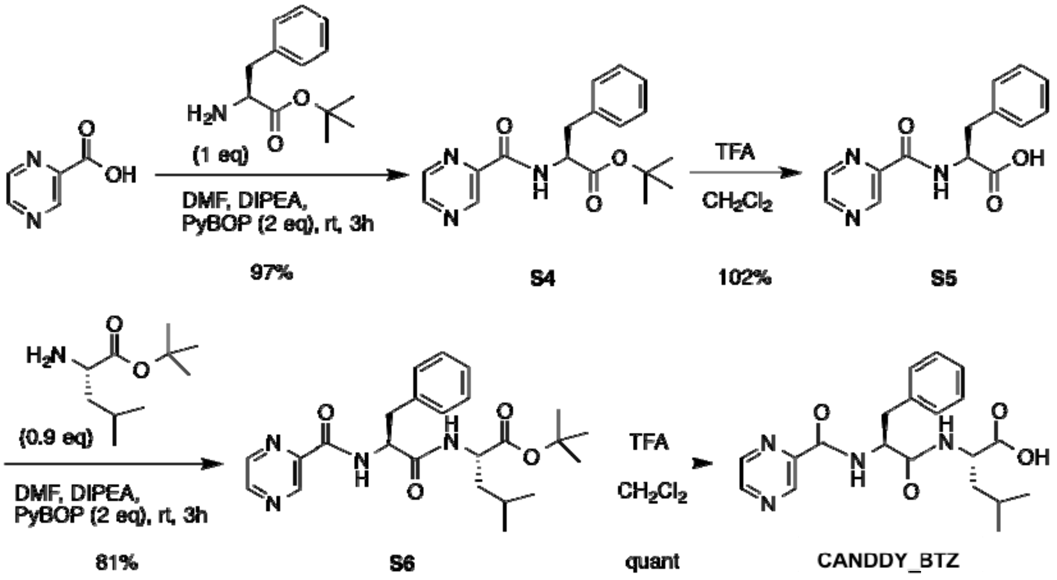

Synthesis of CANDDY_BTZ

To a mixture of a solution of pyrazinecarboxylic acid (253.2 mg, 2.02 mmol) and H-Phe-OtBu · HCl (381.9 mg, 2.00 mmol) in dehydrated *N*,*N*-dimethylformamide (5 mL) and *N*,*N*-diisopropylethylamine (5 mL), PyBOP (2.04 g, 3.84 mmol) was added at room temperature. After stirring for 3 h at the same temperature, a saturated aqueous solution of sodium bicarbonate was added at 0°C. The organic layer was separated and the aqueous layer was extracted with ethyl acetate/hexane (4/1). The combined organic layers were dried over anhydrous sodium sulfate and concentrated *in vacuo*. Further purification was carried out by silica gel column chromatography (eluent: hexane/chloroform; 1/1–0/1, gradient) to produce **S4** (641.5 mg, 97%). To a solution of **S4** (290.2 mg, 0.89 mmol) in dichloromethane (5 mL), trifluoroacetic acid (6 mL) was added at room temperature. After stirring for 1 h at the same temperature, the reaction mixture was concentrated *in vacuo* to produce **S5** (246.3 mg, 102%). To a solution of **S5** (240.8 mg, 0.89 mmol) and H-Leu-OtBu · HCl (183.5 mg, 0.82 mmol) in dehydrated *N*,*N*-dimethylformamide (5 mL) and *N*,*N*-diisopropylethylamine (5 mL), PyBOP (0.99 g, 1.87 mmol) was added at room temperature. After stirring for 3 h at the same temperature, a saturated aqueous solution of sodium bicarbonate was added at 0°C. The organic layer was separated and the aqueous layer was extracted with ethyl acetate. The combined organic layers were dried over anhydrous sodium sulfate and concentrated *in vacuo*. Further purification was carried out by silica gel column chromatography (eluent: hexane/chloroform; 1/1–0/1, gradient) to produce **S6** (317.6 mg, 81%). To a solution of **S6** (180.3 mg, 0.41 mmol) in dichloromethane (5 mL), trifluoroacetic acid (6 mL) was added at room temperature. After stirring for 1 h at the same

temperature, the reaction mixture was concentrated *in vacuo* to produce **CANDDY_BTZ** (173.4 mg, quant.).

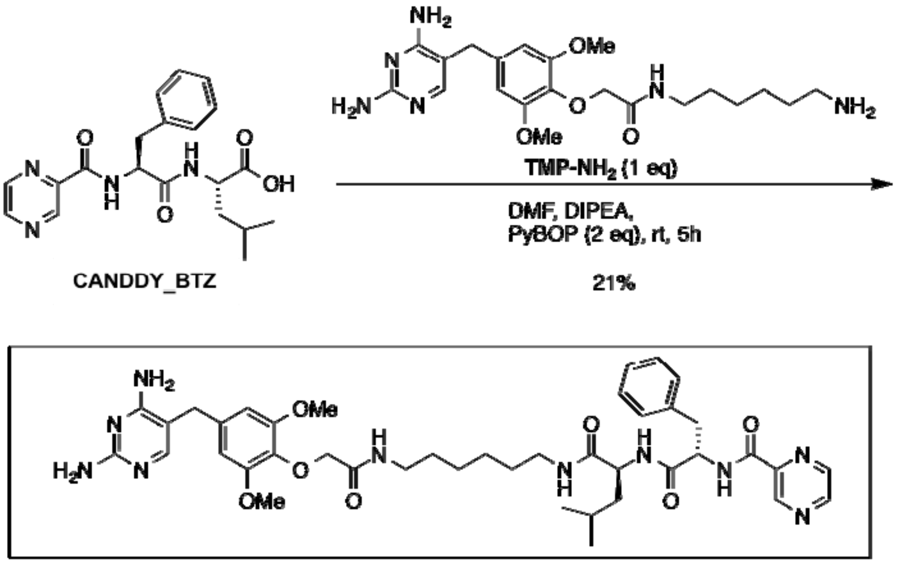

TMP-CANDDY_BTZ (3)

Synthesis of TMP-CANDDY_BTZ (3)

To a solution of **CANDDY_BTZ** (162.3 mg, 0.42 mmol) and TMP-NH_2 (190.3 mg, 0.44 mmol) in dehydrated *N*,*N*-dimethylformamide (5 mL) and *N*,*N*-diisopropylethylamine (10 mL), PyBOP (431.9 mg, 0.83 mmol) was added at room temperature. After stirring for 5 h at the same temperature, a saturated aqueous solution of sodium bicarbonate was added at 0°C. The organic layer was separated and the aqueous layer was extracted with ethyl acetate. The combined organic layers were dried over anhydrous sodium sulfate and concentrated *in vacuo*. Further purification was carried out by silica gel column chromatography (eluent: chloroform/methanol; 10/1–5/1, gradient) to produce **TMP-CANDDY_BTZ** (**3**) (70.5 mg, 21%, isolated yield). A compound for biological assays was purified by PLC (eluent: chloroform/methanol; 10/1). ^{1}H NMR (600 MHz, CD_3OD) δ 0.89 (dd, *J* = 6.6 Hz, 13.2 Hz, 6H), 1.35 (m), 1.45–1.60 (m), 3.12 (dd, *J* = 8.4 Hz, 14.4 Hz), 3.15 (ddd, *J* = 1.8 Hz, 7.2 Hz, 7.2 Hz), 3.28 (m), 3.64 (s, 2H), 3.80 (s, 6H), 4.38 (s, 2H), 4.38 (m), 4.86 (dd, *J* = 5.4 Hz, 8.4 Hz), 6.55 (s, 2H), 7.14–7.26 (m, 5H, Ph), 7.48 (s), 8.62 (dd, *J* = 1.8 Hz. 2.4 Hz), 8.74 (d, *J* = 3.0 Hz), 9.14 (d, *J* = 1.8 Hz); HRMS-FAB (m/z): $[M+H]^+$ calcd for $C_{41}H_{55}N_{10}O_7$, 799.4255; found, 799.4254. HPLC: *t*R = 8.00 min, purity; 98% (λ = 254 nm).

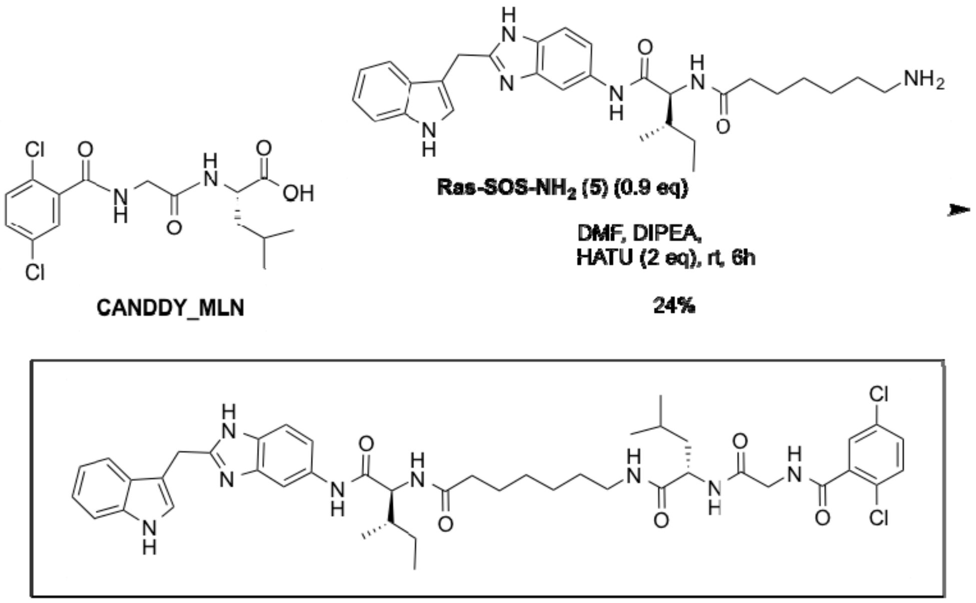

TUS-007

Synthesis of TUS-007

To a solution of **CANDDY_MLN** (52.4 mg, 0.15 mmol) and Ras-SOS-NH_2 (62.4 mg, 0.12 mmol) in dehydrated *N*,*N*-dimethylformamide (4 mL) and *N*,*N*-diisopropylethylamine (4 mL), HATU (114.1 mg, 0.30 mmol) was added at room temperature. After stirring for 6 h at the same temperature, a saturated aqueous solution of sodium bicarbonate was added at 0°C. The organic layer was separated and the aqueous layer was extracted with ethyl acetate. The combined organic layers were dried over anhydrous sodium sulfate and concentrated *in vacuo*. Further purification was carried out by silica gel column chromatography (eluent: chloroform/methanol; 20/1–4/1, gradient) to produce **TUS-007** (25.2 mg, 24%, isolated yield). A compound for biological assays was purified by PLC (eluent: chloroform/methanol; 10/1). ^{1}H NMR (600 MHz, CD_3OD) δ 0.89–0.94 (m, 9H), 0.98 (d, *J* = 6.6 Hz, 3H), 2.26 (m), 1.90 (m), 1.67 (m), 1.59 (m), 1.46 (m), 1.30 (m), 1.24 (m), 3.08–3.19 (m, 2H), 4.35 (s, 2H), 4.41 (dd, *J* = 5.4 Hz, 9.0 Hz), 6.94 (ddd, *J* = 0.6 Hz, 7.2 Hz, 7.2 Hz), 7.07 (ddd, *J* = 1.2 Hz, 7.2 Hz, 7.2 Hz), 7.19 (s), 7.22 (dd, *J* = 2.4 Hz, 6.6 Hz), 7.33 (d, *J* = 7.8 Hz), 7.35–7.42 (m, 4H), 7.57 (dd, *J* = 0.6 Hz, 1.8 Hz), 7.85 (d, *J* = 1.8 Hz); HRMS-FAB (m/z): $[M+H]^+$ calcd for $C_{44}H_{55}Cl_2N_8O_5$, 845.3672; found, 845.3674. HPLC: *t*R = 6.76 min, purity; 98% (λ = 254 nm).

NMR of TUS-007

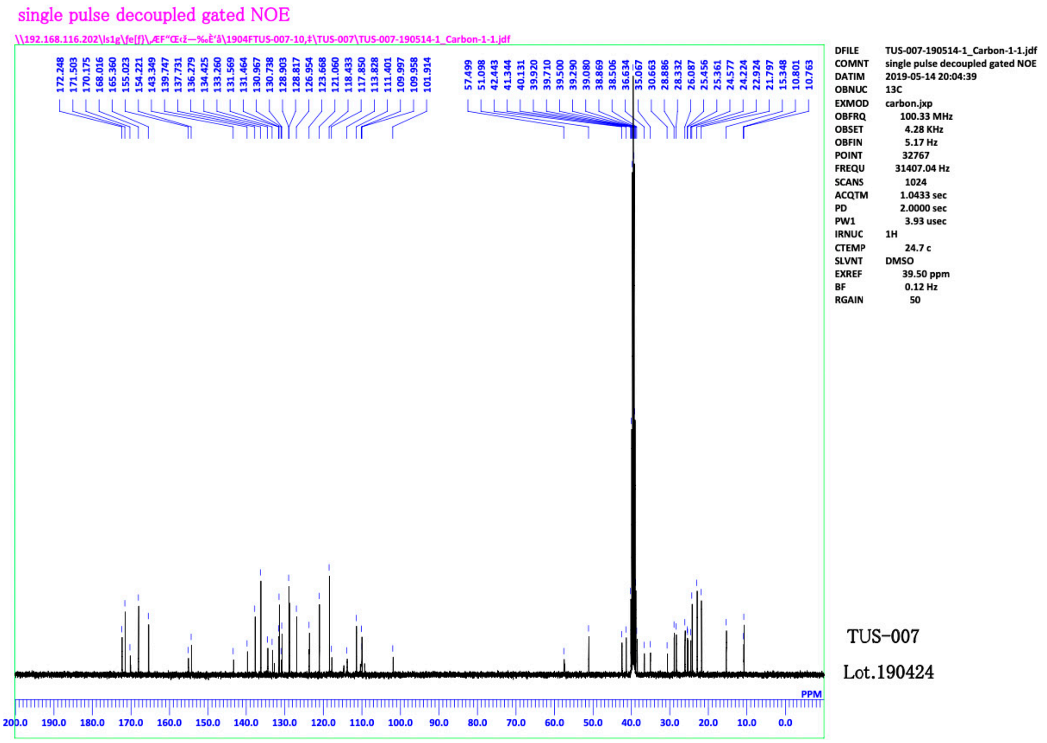

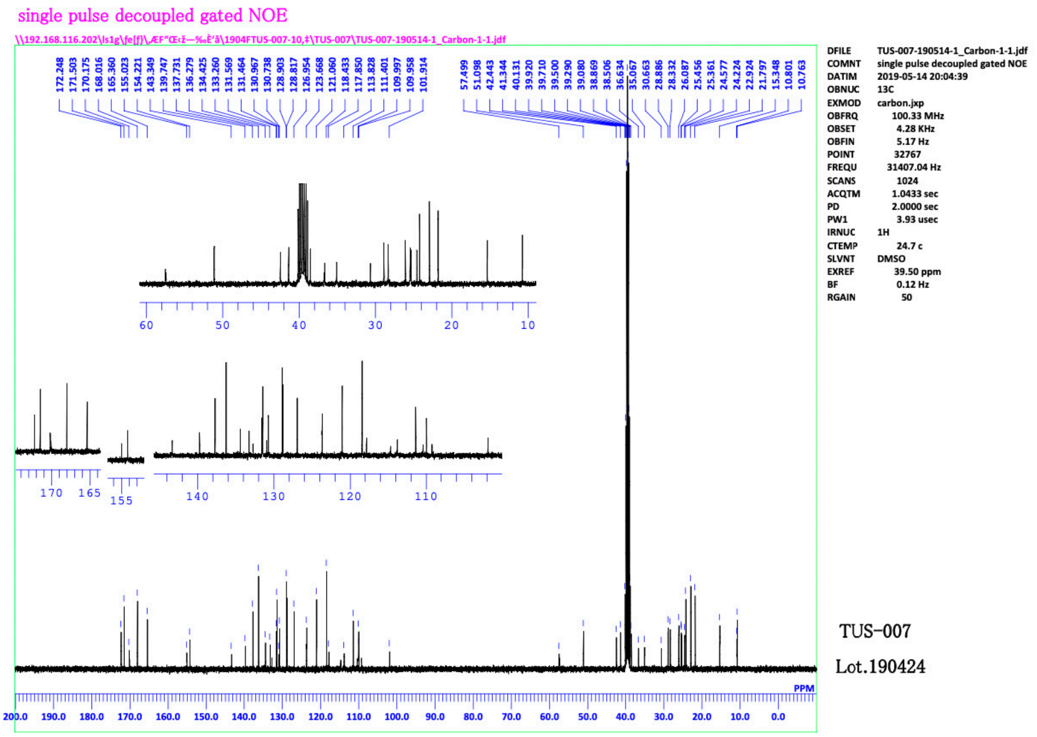

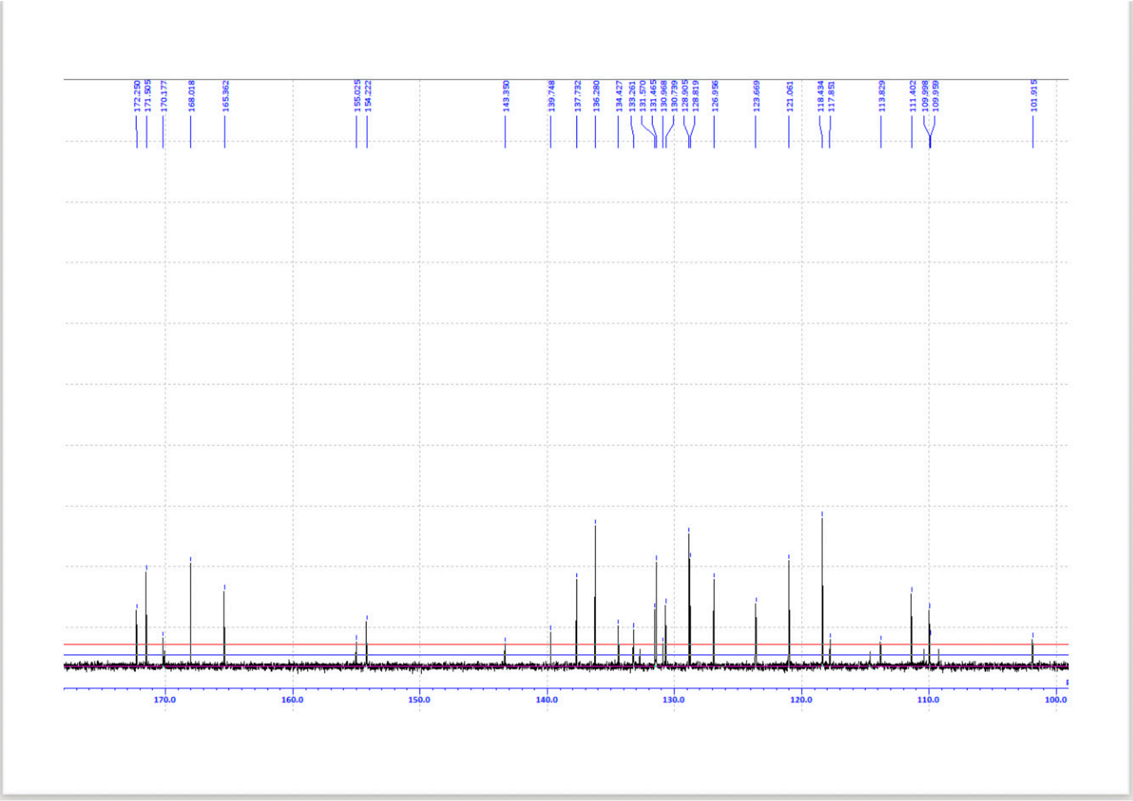

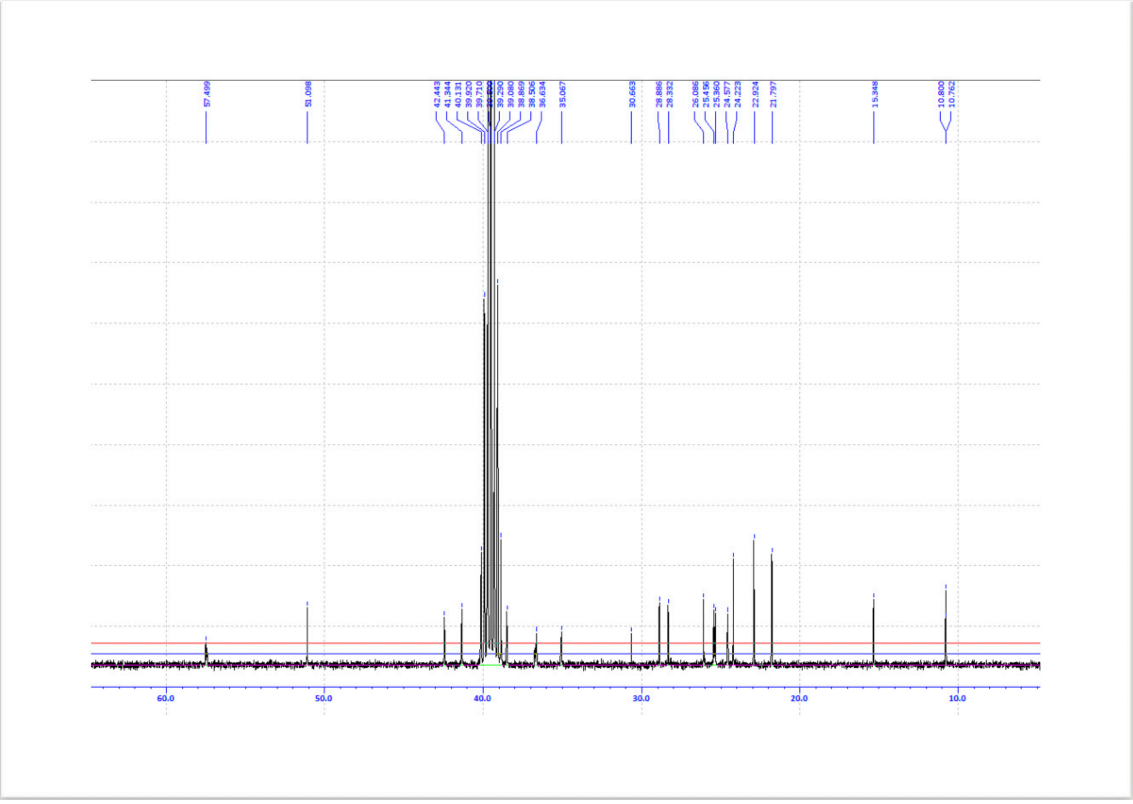

QUANTIFICATION AND STATISTICAL ANALYSIS

FlowJo, Empower, and ImageJ

The data measured by BD FACSCanto II were analyzed using FlowJo (FlowJo LLC). The digital images were obtained by microscopy (BZ-9000, KIYENC). The data measured by HPLC were analyzed using Empower3 (Waters). The individual area of each KRAS stain was identified and measured using ImageJ, and the perimeter to area ratio (KRAS positive area/section) was calculated. The fluorescent images were acquired using a fluorescence microscope (BZ-9000, KIYENC). TUNEL-positive cells were identified and calculated by ImageJ. All reported P values are two-tailed, and for all analyses, p % 0.05 by Student's *t* test is consideredstatistically significant. For descriptive statistics, the group means ± s.e.m. was presented for all relevantdata figures.

Figure S1

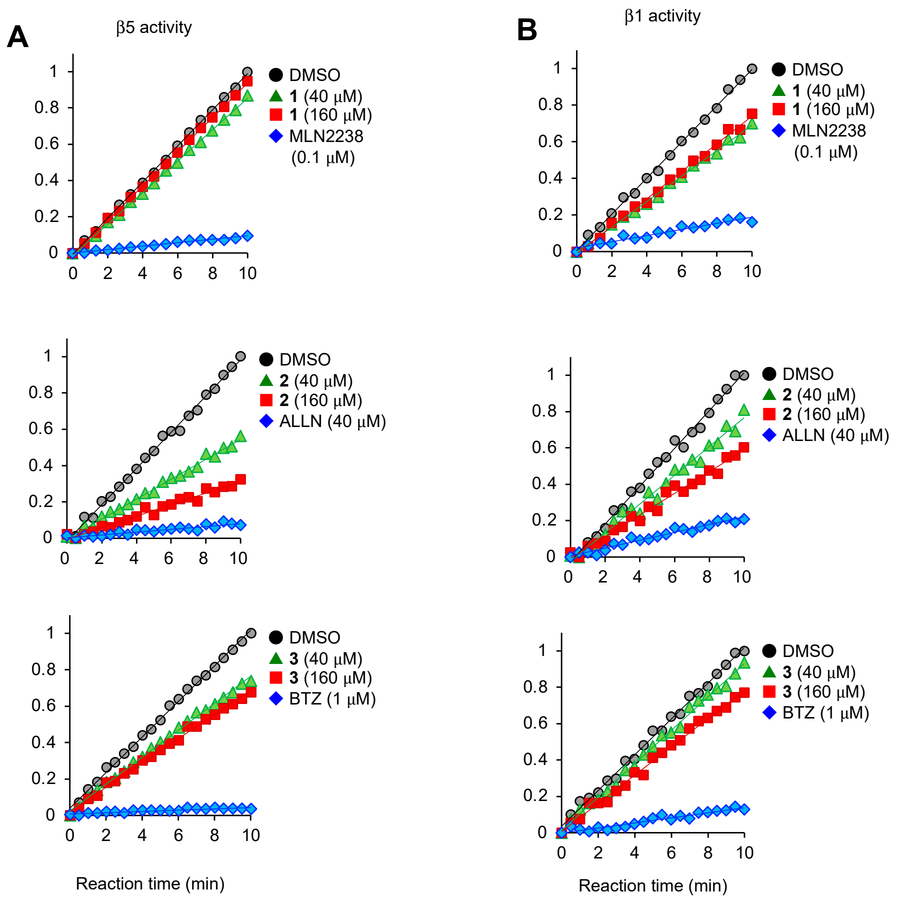

Inhibition of proteasome activity (IC$_{50}$, ∝M).

	β5	β2	β1
TMP-CANDDY_MLN (**1**)	> 160	> 160	> 160
MLN2238	0.0072 (0.00071)	8.9 (2.5)	0.029 (0.014)
TMP-CANDDY_ALLN (**2**)	46.2 (2.0)	> 160	> 160
ALLN	1.0 (0.11)	4.3 (1.0)	9.8 (2.6)
TMP-CANDDY_BTZ (**3**)	> 160	> 160	> 160
BTZ	0.0055 (0.0023)	3.2 (0.54)	0.15 (0.023)

Figure S1. Proteasome Inhibitors Modified as Degradation Tags, Related to Figures 2
Comparison of β5 (A) and β1 (B) proteasomal inhibitory activities of **1**–**3** and each proteasome inhibitor (DMSO, 10 min = 1) (top). IC$_{50}$ values (mM) of the proteasome inhibitory activity of CANDDY molecules and proteasome inhibitors (bottom). SE values are shown in parentheses (mean ± SEM; n = 2).

Figure S2

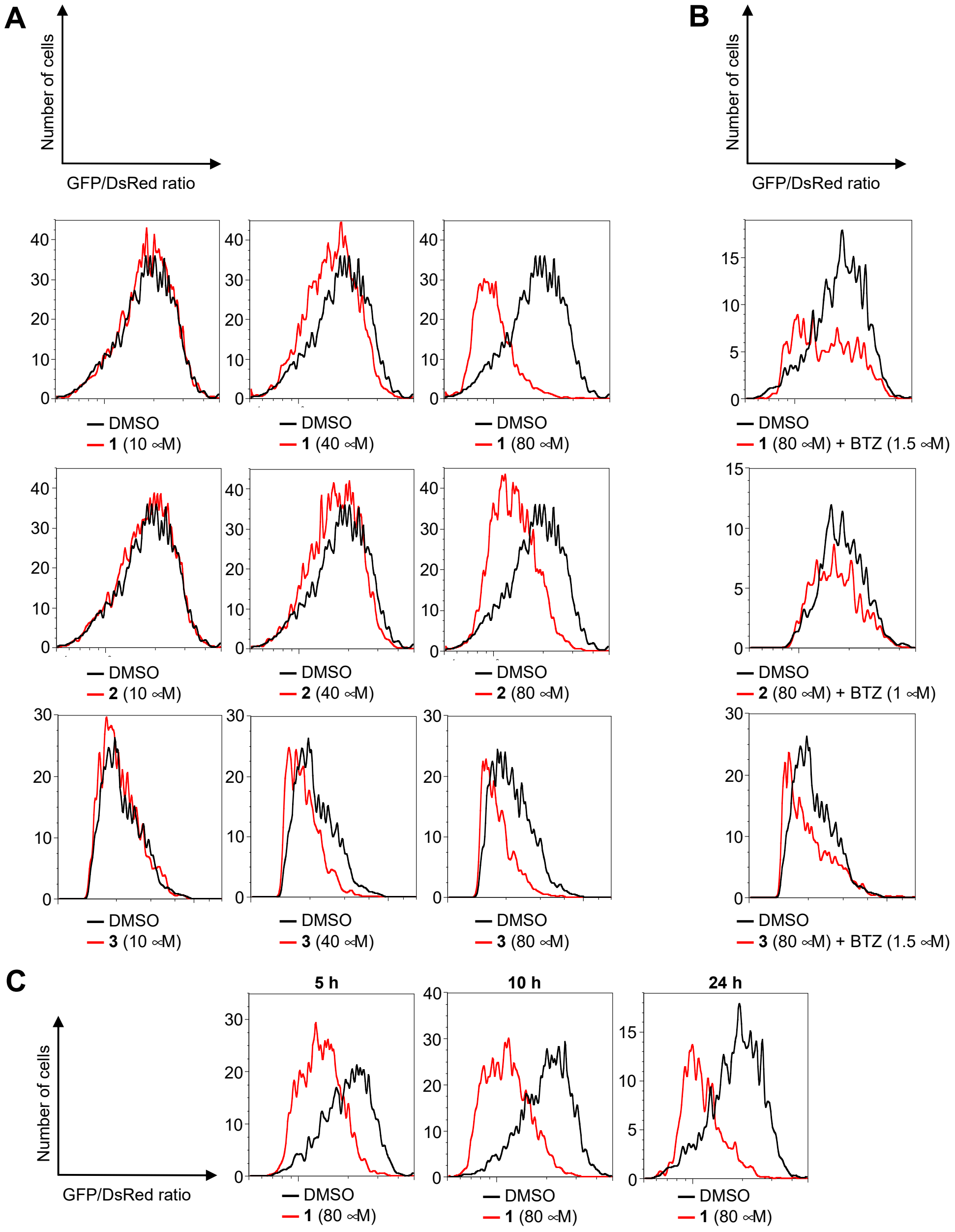

Figure S2. Degradation of ecDHFR Mediated by CANDDY Molecules, Related to Figure 2
HeLa cells co-expressed ecDHFR-HA-GFP and DsRed from a bicistronic construct. Target-expressing HeLa cells were treated with **1–3** for 24 h.
(A) **1–3** dose-dependently degraded ecDHFR-HA-GFP protein levels as determined by flow cytometry.
(B) Degradation of ecDHFR by **1–3** was rescued by the proteasome inhibitor bortezomib (BTZ).
(C) HeLa cells co-expressed ecDHFR-HA-GFP and DsRed from a bicistronic construct. Target-expressing HeLa cells were treated with a CANDDY molecule (**1**). **1** degraded ecDHFR-HA-GFP protein levels in a time-dependent manner as determined by flow cytometry.

Figure S3

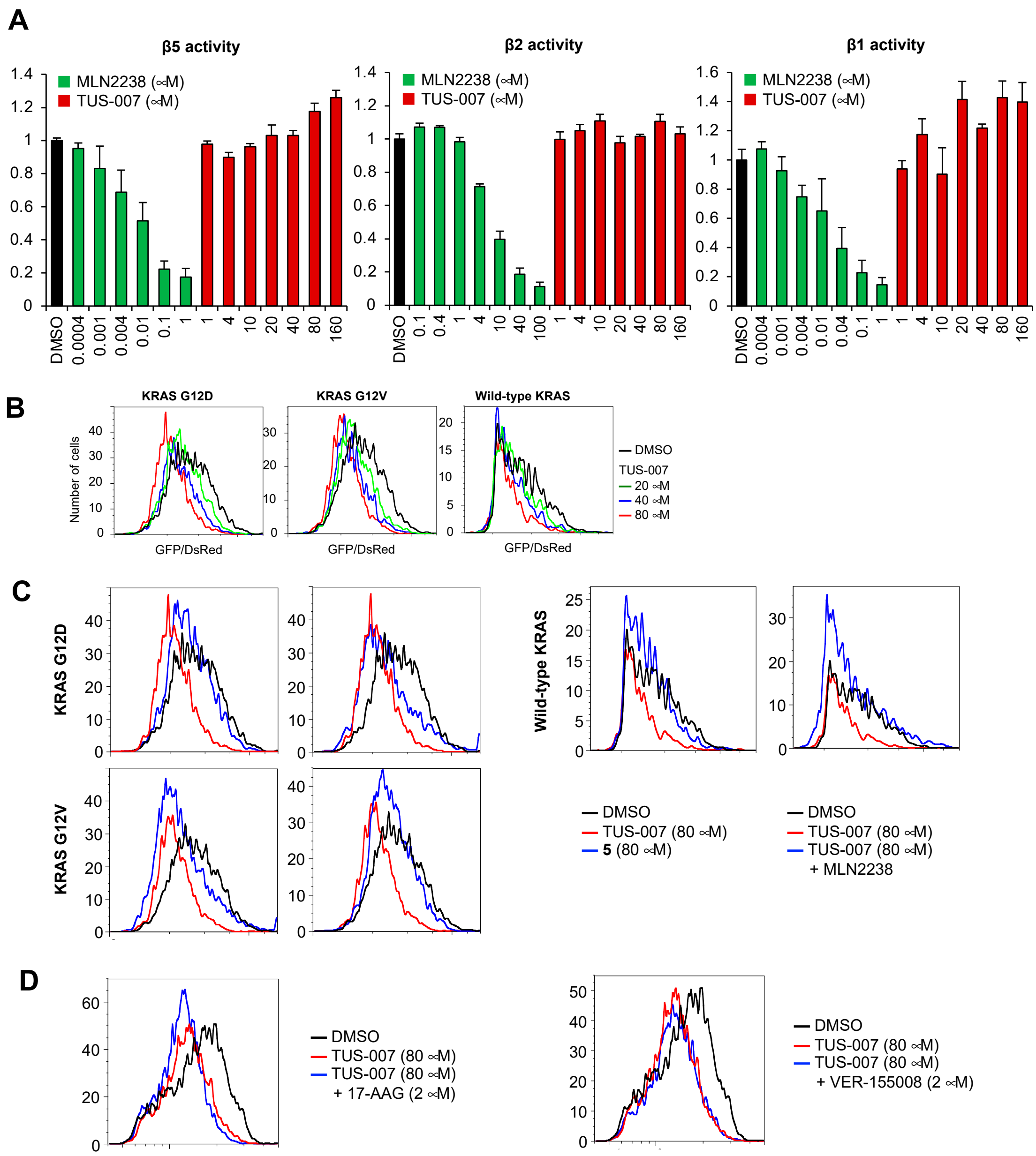

Figure S3. Proteasomal Inhibitory Activities of TUS-007 and Degradation of KRAS by TUS-007, Related to Figure 3
(A) Chymotrypsin-like (β5) , trypsin-like (β2) and caspase-like (β1) activities of the proteasome were monitored by Suc-LLVY-AMC (β5 substrate), Bz-VGR-AMC (β2 substrate) and Z-LLE-AMC (β1 substrate), respectively. AMC fluorescence was monitored by a plate reader with excitation and emission filters of 360 nm and 460 nm, respectively (DMSO, 30 min = 1) (mean ± SEM; n = 2–3 (B) Degradation of KRAS G12D, KRAS G12V and wild-type KRAS proteins by TUS-007. KRAS-HA-GFP and DsRed-expressing HeLa cells were treated with TUS-007 for 24 h and then analyzed by flow cytometry. (C) Target-expressing HeLa cells were treated with TUS-007 and RAS-SOS-NH2 (5) for 24 h and then analyzed by flow cytometry. Degradation of mutant (G12D and G12V) and wild-type KRAS proteins by TUS-007 was rescued by treatment with the proteasome inhibitor MLN2238 (1.5 mM). (D) Chaperone-independent proteasomal mutant KRAS degradation. KRAS G12D-HA-GFP and DsRed-expressing HeLa cells were co-treated with TUS-007 and 17-AAG (HSP90 inhibitor, 2 ∝M), or VER-155008 (HSP70 inhibitor, 2 ∝M) for 24 h and then analyzed by flow cytometry.

Figure S4

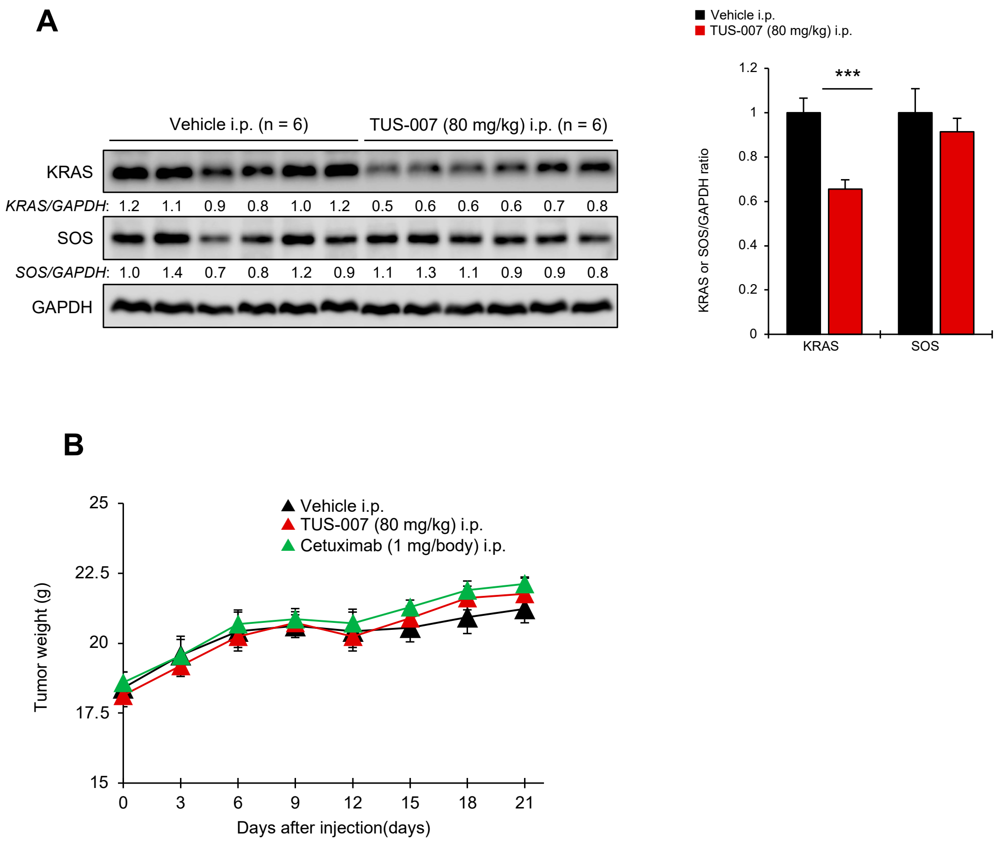

Figure S4. Targets degradation in SW620 and SW1990 Orthotopically-Transplanted Mice, and Toxicity Test of TUS-007, Related to Figure 4.
(A) Immunoblotting data and quantification of KRAS G12V and SOS (negative control) proteins by immunoblotting using tumor lysates from day 21 in Figure 4D and E (mean ± SEM; n = 6). ***p < 0.001 (Student's t test). (B) Body weight changes of SW620 subcutaneously-transplanted mice treated with vehicle, TUS-007, or cetuximab in Figure 4D and E (mean ± SEM; n = 6–9).

Figure S5

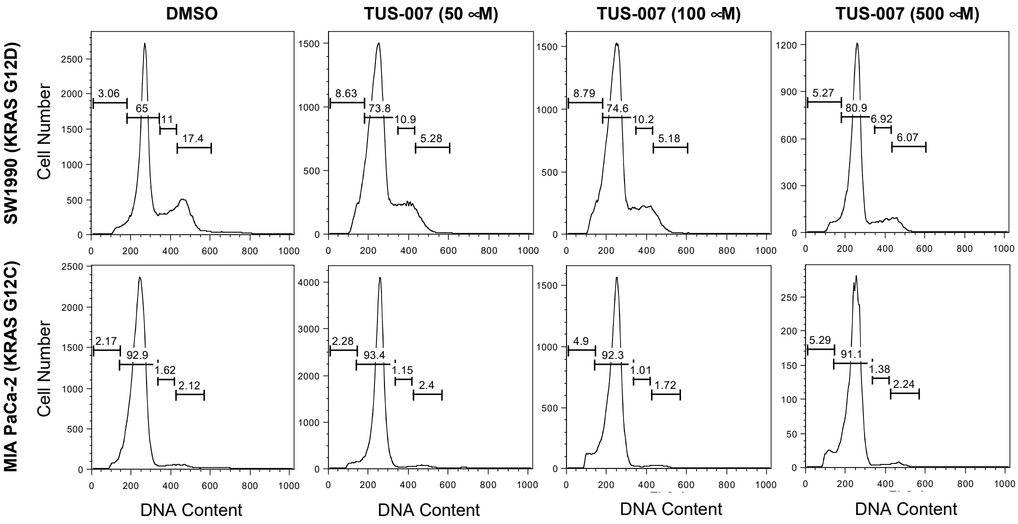

Figure S5. Cell Cycle Regulation, Apoptosis Induction, by TUS-007, Related to Figure 5
SW1990 and MIA PaCa-2 cells were treated with TUS-007 for 72 h and then detected by flow cytometry. Flow cytometry data.

Figure S6

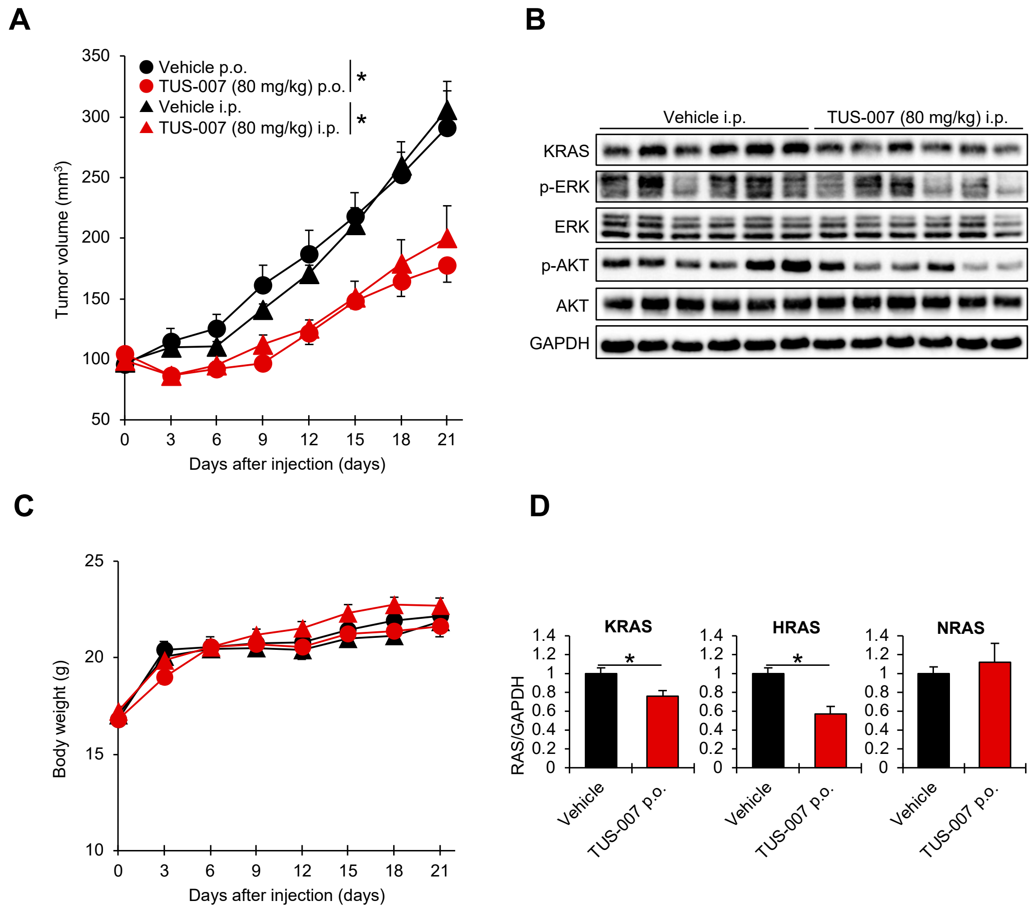

Figure S6. KRAS-Mutant Pancreatic Cancer Tumor Suppression, Related to Figure 6
(A) Tumor volume of vehicle-treated SW1990 subcutaneously-transplanted mice and mice treated with TUS-007 (mean ± SEM; *n* = 6–9). Administration was undertaken every three days. *p < 0.05 (Student's *t* test). (B) Tumor weight at 21 days after injection in (A). (C)) Body weight changes of SW1990 subcutaneously-transplanted mice treated with vehicle or TUS-007 in (A) (mean ± SEM; *n* = 6–9). (D) Effect of TUS-007 against RAS family in pancreatic tissue of (B). * p < 0.05 (Student's *t* test).

Figure S7

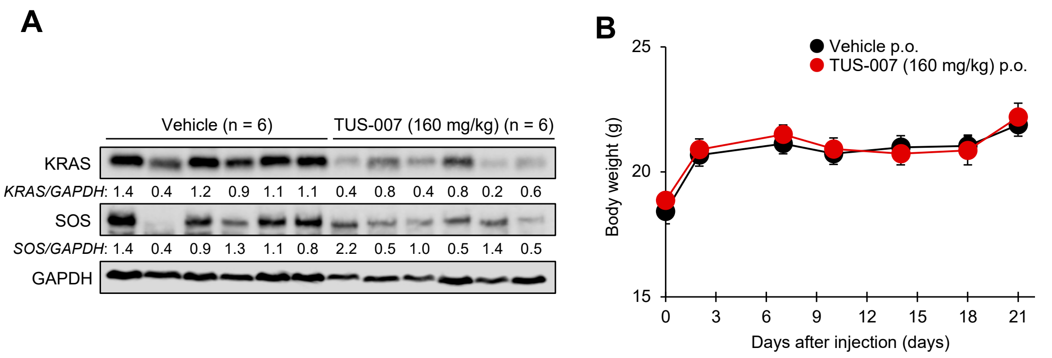

Figure S7. Targets degradation in SW620 and SW1990 Orthotopically-Transplanted Mice, and Toxicity Test of TUS-007, Related to Figure 6.
(A) Degradation of KRAS protein in SW1990-luc tumor. Immunoblotting data of Figure 6. (b) Body weight change of mouse in Figure 6 (mean ± SEM; *n* = 6).
